# The E3 ubiquitin ligase Nedd4L preserves skeletal muscle stem cell quiescence by inhibiting their activation

**DOI:** 10.1016/j.isci.2024.110241

**Published:** 2024-06-11

**Authors:** Darren M. Blackburn, Korin Sahinyan, Aldo Hernández-Corchado, Felicia Lazure, Vincent Richard, Laura Raco, Gabrielle Perron, René P. Zahedi, Christoph H. Borchers, Christoph Lepper, Hiroshi Kawabe, Arezu Jahani-Asl, Hamed S. Najafabadi, Vahab D. Soleimani

**Affiliations:** 1 Department of Human Genetics, McGill University, 3640 rue University, Montréal, QC H3A 0C7, Canada; 2 Lady Davis Institute for Medical Research, Jewish General Hospital, 3755 Chemin de la Côte- Sainte-Catherine, Montréal, QC H3T 1E2, Canada; 3 Segal Cancer Proteomics Centre, Lady Davis Institute, Jewish General Hospital, McGill University, Montréal, QC H3T 1E2, Canada; 4 Manitoba Centre for Proteomics and Systems Biology, Winnipeg, MB R3E 3P4, Canada; 5 Department of Internal Medicine, University of Manitoba, Winnipeg, MB R3E 3P4, Canada; 6 Department of Biochemistry and Medical Genetics, University of Manitoba, Winnipeg, MB R3E 0J9, Canada; 7 Gerald Bronfman Department of Oncology, Lady Davis Institute for Medical Research, Jewish General Hospital, Montréal, QC H3T 1E2, Canada; 8 Division of Experimental Medicine, McGill University, Montréal, QC H4A 3J1, Canada; 9 Department of Pathology, McGill University, Montréal, QC H3A 2B4, Canada; 10 Department of Physiology & Cell Biology, College of Medicine, The Ohio State University, Columbus, OH, USA; 11 Department of Molecular Neurobiology, Max Planck Institute of Experimental Medicine 37075 Göttingen, Germany; 12 Department of Cellular and Molecular Medicine and University of Ottawa Brain and Mind Research Institute, 451 Smyth Rd, Ottawa, ON K1H 8M5, Canada; 13 Department of Biochemistry, Microbiology & Immunology, University of Ottawa, 451 Smyth Rd, Ottawa, ON K1H 8M5, Canada

**Keywords:** natural sciences, biological sciences, biochemistry, cell biology, stem cells research

## Abstract

Adult stem cells play a critical role in tissue repair and maintenance. In tissues with slow turnover, including skeletal muscle, these cells are maintained in a mitotically quiescent state yet remain poised to re-enter the cell cycle to replenish themselves and regenerate the tissue. Using a panomics approach we show that the PAX7/NEDD4L axis acts against muscle stem cell activation in homeostatic skeletal muscle. Our findings suggest that PAX7 transcriptionally activates the E3 ubiquitin ligase Nedd4L and that the conditional genetic deletion of Nedd4L impairs muscle stem cell quiescence, with an upregulation of cell cycle and myogenic differentiation genes. Loss of Nedd4L in muscle stem cells results in the expression of doublecortin (DCX), which is exclusively expressed during their *in vivo* activation. Together, these data establish that the ubiquitin proteasome system, mediated by Nedd4L, is a key contributor to the muscle stem cell quiescent state in adult mice.

## Highlights

• The E3 ubiquitin ligase Nedd4L maintains muscle stem cells in a quiescent state
• Nedd4L-cKO muscle stem cells express higher levels of MyoD, phospho-S6 kinase, and DCX
• Entry into shallow state of quiescence does not impact chromatin accessibility
• Nedd4L expression is positively regulated by Pax7

## Graphical abstract

**Figure:**
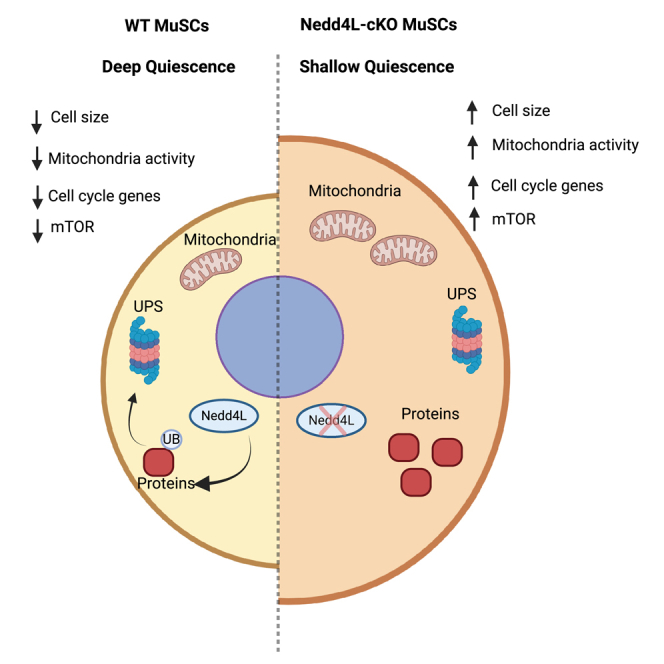

## Introduction

Tissue-specific stem cells are essential for the maintenance and regeneration of the tissue in which they reside. Postnatally, adult skeletal muscle is maintained and repaired by a rare population of muscle stem cells (MuSCs), also known as satellite cells.^1^^,^^2^ In the absence of trauma or injury, these cells typically exist in a quiescent state and are marked by the expression of the transcription factor Pax7.^3^^,^^4^ However, they can quickly respond in the event of an injury to the muscle and activate to re-enter the cell cycle. Once activated, MuSCs will proliferate, differentiate, and self-renew, thus repairing the damaged muscle and restoring tissue homeostasis.^2^

Maintenance of quiescence is crucial to prevent the exhaustion of the adult stem cell pool to precocious activation and differentiation. Previous studies on skeletal muscles have demonstrated that stem cell quiescence is a complex and dynamic state that is actively maintained.^5^^,^^6^^,^^7^^,^^8^ For example, the state of G_alert_ was first described in MuSCs^9^ in which the stem cells are no longer in deep quiescence but have not yet fully transitioned into activation and the cell cycle. Differences in the depth of quiescence were also identified in other adult stem cells, including neuronal stem cells (NSCs) and hematopoietic stem cells (HSCs).^10^^,^^11^^,^^12^ Characteristics of G_alert_ include larger cell size, higher mitochondria content, increased translation of mRNA, and a more rapid cell division at the time of activation.^9^ Multiple signaling pathways have been identified to facilitate the transition of MuSCs into G_alert_, including the activation of mammalian target of rapamycin (mTOR),^9^ the mitochondrial protein OPA1,^13^ and the transcription factor GLI3.^14^ The G_alert_ state has also been shown to result from an injury in a distant tissue.^15^ Together, these studies suggest that transition of quiescent adult stem cells into the cell cycle constitutes a continuum with potentially numerous checkpoints along the way.

In adult stem cells, several mechanisms have been identified that poise the cells for activation while simultaneously maintaining quiescence.^16^ Of note, in MuSCs, it is known that there is a large amount of bivalent chromatin, possessing both permissive and repressive marks, indicating that these genes can be quickly transcribed in the case of activation.^17^ MuSCs also have low mRNA content due primarily to transcriptional repression from the lack of RNA polymerase II phosphorylation.^18^^,^^19^ Translational control of the produced mRNA can play a role in the poised state of MuSCs. It has been previously reported that both Myf5 and MyoD1 transcripts can be sequestered in quiescent MuSCs, preventing their translation.^8^^,^^20^ mRNA degradation by miRNAs is also a well-described mechanism for the maintenance of quiescence, with these miRNAs quickly downregulated in the case of activation.^21^^,^^22^

The ubiquitin-proteasome system (UPS) is responsible for the targeted degradation of proteins. Proteins that are destined for degradation will be K48 polyubiquitinated. This process is composed of a chain of enzymatic reactions executed by the E1 ubiquitin-activating enzyme, an E2 ubiquitin transporter, and an E3 ubiquitin ligase.^23^ The role of the UPS in the maintenance of MuSC quiescence is not well understood; however, the disruption of the UPS via the genetic deletion of *Rpt3*, a component of the proteasome, has been shown to cause the MuSCs to become apoptotic.^24^ In HSCs undergoing activation, select E3 ubiquitin ligases have been shown to decrease in expression,^25^^,^^26^^,^^27^ which could suggest a role for these enzymes in the regulation of adult stem cell quiescence. A recent study has shown that the Skp1-Cul1-F-box protein ubiquitin ligase complex maintains MuSC quiescence.^28^

Key members of the UPS are the E3 ubiquitin ligases. These enzymes are responsible for tagging the proteins with ubiquitin, targeting them to the proteasome for degradation. Neural precursor cell expressed developmentally downregulated 4-like (Nedd4L) is an E3 ubiquitin ligase that is part of the HECT family.^29^ Nedd4l is known to target several membrane proteins, such as the amiloride-sensitive epithelial Na+ channel (ENaC), TGFBR1, and voltage-gated sodium channels (Na_v_s).^30^^,^^31^^,^^32^^,^^33^^,^^34^ It is also known to act as a tumor suppressor in human cancers, with a decrease in its expression correlating with worse prognosis.^29^^,^^35^^,^^36^^,^^37^^,^^38^ A previous study has demonstrated that Nedd4, the founding member of the Nedd4 family of which Nedd4l is part of, could target Pax7 for degradation and drive the MuSCs to activate.^39^ It is important to note that although the two enzymes are structurally similar, they are functionally distinct. The Nedd4 enzyme is a known oncogene, whereas Nedd4L has the opposing function as a tumor suppressor.^29^^,^^35^^,^^40^^,^^41^

The role of Nedd4l in the context of MuSC quiescence has not been investigated. In the present study, we report a central role for Nedd4l in maintaining MuSC quiescence downstream of PAX7. Our data suggest that the PAX7/NEDD4L axis prevents the transition of MuSC from quiescence into activation. Importantly, genetic deletion of Nedd4l results in the MuSCs exit from quiescence, leading to their entry into a cellular state that is reminiscent of G_alert_.

## Results

### Pax7 transcriptionally regulates the expression of the E3 ubiquitin ligase Nedd4L

**Figure 1:**
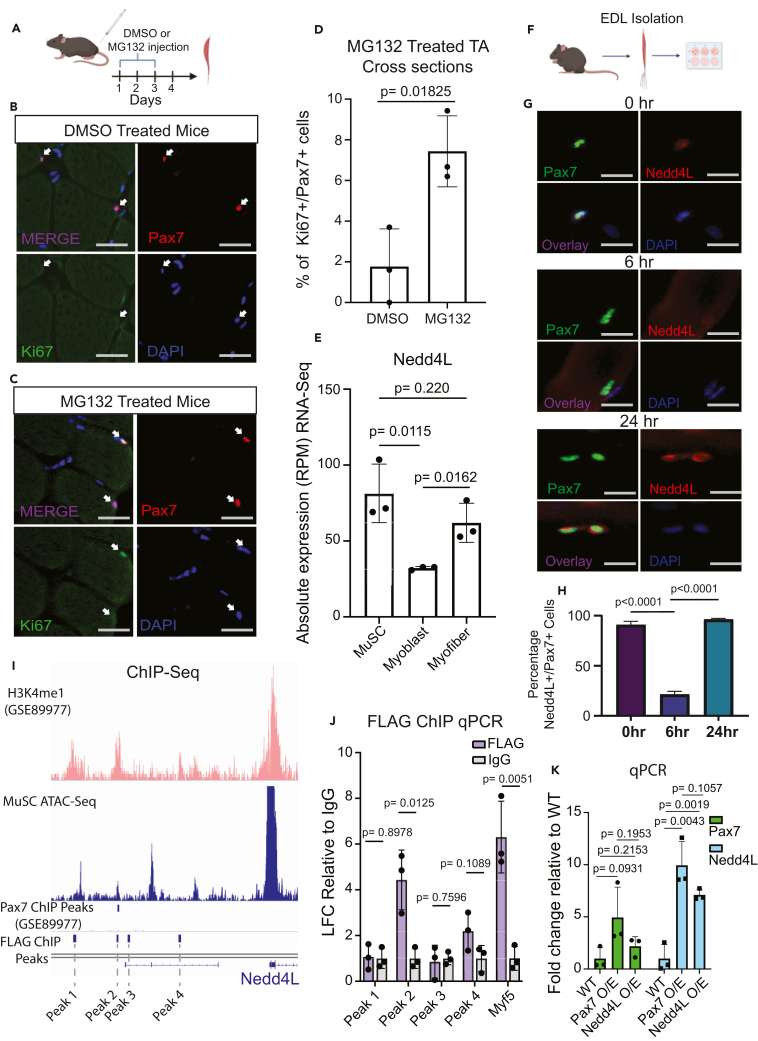
Pax7 transcriptionally regulates the expression of the E3 ubiquitin ligase Nedd4L (A) Diagram showing the TA of WT mice were intramuscularly injected with either DMSO or MG132 for 3 days, generated with Biorender.com. (B) Representative immunofluorescent staining for Pax7 and Ki67 of cross-sections from TA muscle treated with DMSO control. (C) Representative immunofluorescent staining for Pax7 and Ki67 of cross-sections from TA muscle treated with MG132. (D) Bar graph representing the percentage of double-positive Pax7^+^ and Ki67^+^ MuSCs. *n* = 3; two-tailed t test, data represented as mean ± SD. (E) Bar graph of the absolute expression from RNA-seq of Nedd4L from freshly isolated MuSCs, myoblasts 3 days post-isolation, and single EDL myofibers. Single myofiber data are retrieved from the GEO GSE138591.^42^*n* = 3; two-tailed t test, data presented as mean ± SD. (F) Diagram of EDL isolation from mice, generated with Biorender.com. (G) Immunofluorescent staining of EDL myofibers for Pax7 and Nedd4L at 0, 6, and 24 h post-isolation and staining for Myogenin and Nedd4L at 60 h post-isolation. (H) Bar graph representing the percentage of Nedd4L^+^/Pax7^+^ cells on EDL myofibers 0 h, 6 h, and 24 h post-isolation. Data are presented as mean ± SD. *n* = 3; two-tailed t test, data presented as mean ± SD. (I) IGV tracks of the enhancer region upstream of the Nedd4L promotor. The H3K4me1 tracks (GSM2394788) were retrieved from the GEO GSE89977,^43^ and the Pax7 ChIP-Seq Peaks were called from the signal of GSM2394784 (+dox) by using GSM2394785 (−dox) as background.^43^ The Pax7-FLAG ChIP-seq peaks were generated from primary myoblasts that were overexpressing Pax7-FLAG. (J) Bar graph of the FLAG ChIP-qPCR of Pax7-FLAG overexpressing primary myoblasts, representing the LFC enrichment relative to the immunoglobulin G (IgG) control of the four nearest PAX7 peaks as determined by the FLAG ChIP-seq with Myf5 as a positive control. *n* = 3; two-tailed t test, data presented as mean ± SD. (K) qPCR of Pax7 and Nedd4L of WT, Pax7-FLAG overexpressing, and Nedd4L overexpressing primary myoblasts. *n* = 3; two-tailed t test, data presented as mean ± SD.

To confirm that the UPS is essential for maintaining MuSC quiescence, we injected tibialis anterior (TA) muscles of mice with MG132, a general inhibitor of the proteasome system, for 3 days (Figure 1A). Analysis of the TA cross-sections showed that the MuSCs in the treated TAs were more prone to exit quiescence as shown by the expression of the proliferation marker Ki67 (Figures 1A–1D). RNA sequencing (RNA-seq) analysis of freshly sorted MuSCs and primary myoblasts showed that 252 E3 ubiquitin ligases are expressed at a threshold of at least 100 base counts. An unbiased analysis of the E3 ubiquitin ligases whose expression change during the transition from quiescent MuSCs to cultured primary myoblasts, revealed that 120 E3 ligases exhibit a significant change in expression (Figure S1). We chose to focus on Nedd4L, as it is highly expressed in quiescent MuSCs and highly conserved in mammals. Nedd4L is part of the Nedd4 family of E3 ubiquitin ligases. Although Nedd4 is very highly expressed in muscle tissue, its expression is relatively static throughout the myogenic pathway and is not differentially expressed between quiescent MuSCs and myoblasts (Figure S1B). Through RNA-seq, we see that Nedd4L is most highly expressed in quiescent MuSCs, with a drop in expression in the myoblast stage and an increase in expression in the terminally differentiated myofibers (Figure 1E). Additionally, the expression profile of Nedd4L is highly dynamic in myofiber-associated MuSCs during *in vitro* culture (Figures 1F–1H), with the majority of quiescent MuSCs expressing Nedd4L and quickly losing that expression 6 h post-isolation only to regain the protein at the 24-h time point.

Pax7 is a critical transcription factor for MuSC function and is highly expressed in quiescent MuSCs.^3^^,^^44^ Therefore, we analyzed independent chromatin immunoprecipitation sequencing (ChIP-seq) datasets for potential regulation of Nedd4L by Pax7 in muscle cells. ChIP-seq data from Lilja et al. indicate that there is a proximal enhancer region upstream from the Nedd4L promoter^43^ (Figure 1I). The study by Lilja et al. used a doxycycline system to express Pax7 in pluripotent stem cells, and from their Pax7 ChIP-seq we see that there is a Pax7-binding peak at these enhancers,^43^ which is confirmed with our own Pax7-FLAG ChIP-seq in myoblasts (Figure 1I). These enhancers are also accessible in MuSCs as seen by our ATAC-Seq data on freshly sorted MuSCs (Figure 1I). Together, these data suggest that Pax7 regulates Nedd4L expression. To further confirm this finding, we generated myoblasts stably over-expressing Pax7-FLAG protein and performed a FLAG tag ChIP-qPCR. We observed that there is enrichment for PAX7 binding to these enhancer elements (Figure 1J). Next, with real-time qPCR of the RNA from the Pax7-FLAG overexpressing myoblasts we confirmed the upregulation of Nedd4L (Figure 1K). These data indicate that Pax7 positively regulates the expression of Nedd4L.

### Genetic deletion of Nedd4L results in the loss of quiescence-associated genes in MuSCs

To further elucidate the role of Nedd4L in MuSC function, we used a genetic mouse model where Nedd4L is specifically deleted in MuSCs using a Cre-loxP system. In this model, exon 15 of *NEDD4L*, which codes for part of the catalytic domain of the protein, is flanked by *loxP* sites and deleted in cells expressing Pax7 (Figure S2A). The gene is still being transcribed, but without Exon 15 it is catalytically inactive^31^ (Figures S2A and S2B).

**Figure 2:**
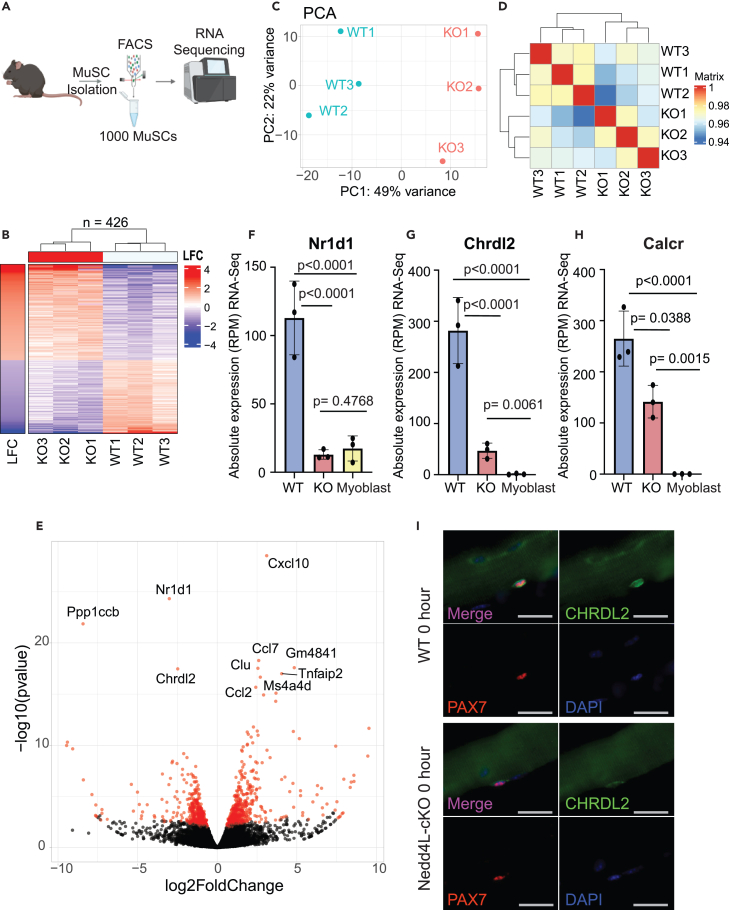
Genetic deletion of Nedd4L results in the loss of quiescence-associated genes in MuSCs (A) Schematic of the isolation of MuSCs and RNA-seq, generated with Biorender.com. *n* = 3 male mice per condition, aged 5–6 weeks. (B) Heatmap of differentially expressed genes between WT and Nedd4L-cKO MuSCs. Threshold is adjusted *p* value ≤0.05, LFC ≥1, RPM ≥5. (C) PCA plot of WT and Nedd4L-cKO MuSCs. (D) Pearson correlation of the WT and Nedd4L-cKO MuSCs. (E) Volcano plot of WT vs. Nedd4L-cKO transcripts. (F) Bar graph of the absolute expression (RPM) of *Nr1d1* in WT and Nedd4L-cKO MuSCs and in WT myoblasts. *n* = 3; two-tailed t test, data presented as mean ± SD. (G) Bar graph of the absolute expression (RPM) of *Chrdl2* in WT and Nedd4L-cKO MuSCs and in WT myoblasts. *n* = 3; two-tailed t test, data presented as mean ± SD. (H) Bar graph of the absolute expression (RPM) of *Calcr* in WT and Nedd4L-cKO. *n* = 3; two-tailed t test, data presented as mean ± SD. (I) Immunofluorescence of Pax7 and Chrdl2 in MuSCs associated to freshly isolated EDL myofibers. Scale bar: 25 μm.

We analyzed the transcriptome of freshly isolated MuSCs by fluorescence-activated cell sorting (FACS) from wild-type (WT) and Nedd4L-cKO mice using SMART-seq (Figure 2A). From this analysis, we found that 426 genes were differentially expressed between WT and Nedd4L-cKO mice (Figure 2B). Principal-component analysis (PCA) shows a clear separation of WT and Nedd4L-cKO MuSCs, and with Pearson correlation we note that Nedd4L-cKO cells are more similar to one another than their WT counterparts (Figures 2C–2E). Interestingly, some of the most significantly downregulated genes, such as *NR1D1*, *CHRDL2,* and *CALCR*, are known markers of quiescence (Figures 2F–2H). *NR1D1* is known to inhibit proliferation and myogenesis^45^ and is greatly reduced in expression in myoblasts compared to MuSCs (Figure 2F). *CALCR* is a receptor that has been determined to be important for the maintenance of MuSC quiescence.^46^^,^^47^ *CHRDL2* is known to be a marker of quiescence^48^^,^^49^ and whose expression is completely lost in myoblasts (Figure 2G). The loss of *Chrdl2* transcript in the Nedd4L-cKO correlates with a reduction in its protein levels in freshly isolated extensor digitorum longus (EDL)-myofiber-associated MuSCs of Nedd4L-cKO mice (Figure 2I). Further analysis of the transcriptomic data shows that of the top 100 most differentially expressed genes, the majority are upregulated (Figure S2C). We questioned whether this was due to changes in RNA stability, by using estimated RNA turnover as a proxy where the ratio of intronic and exonic read counts are compared, and found that only 10 genes have a change in their stability. Interestingly, two of the genes that had a decrease in their RNA stability are *NR1D1* and *PAX7* (Figure S2D), which may explain the loss of *Nr1d1* expression in the Nedd4L-cKO MuSCs.

**Figure 3:**
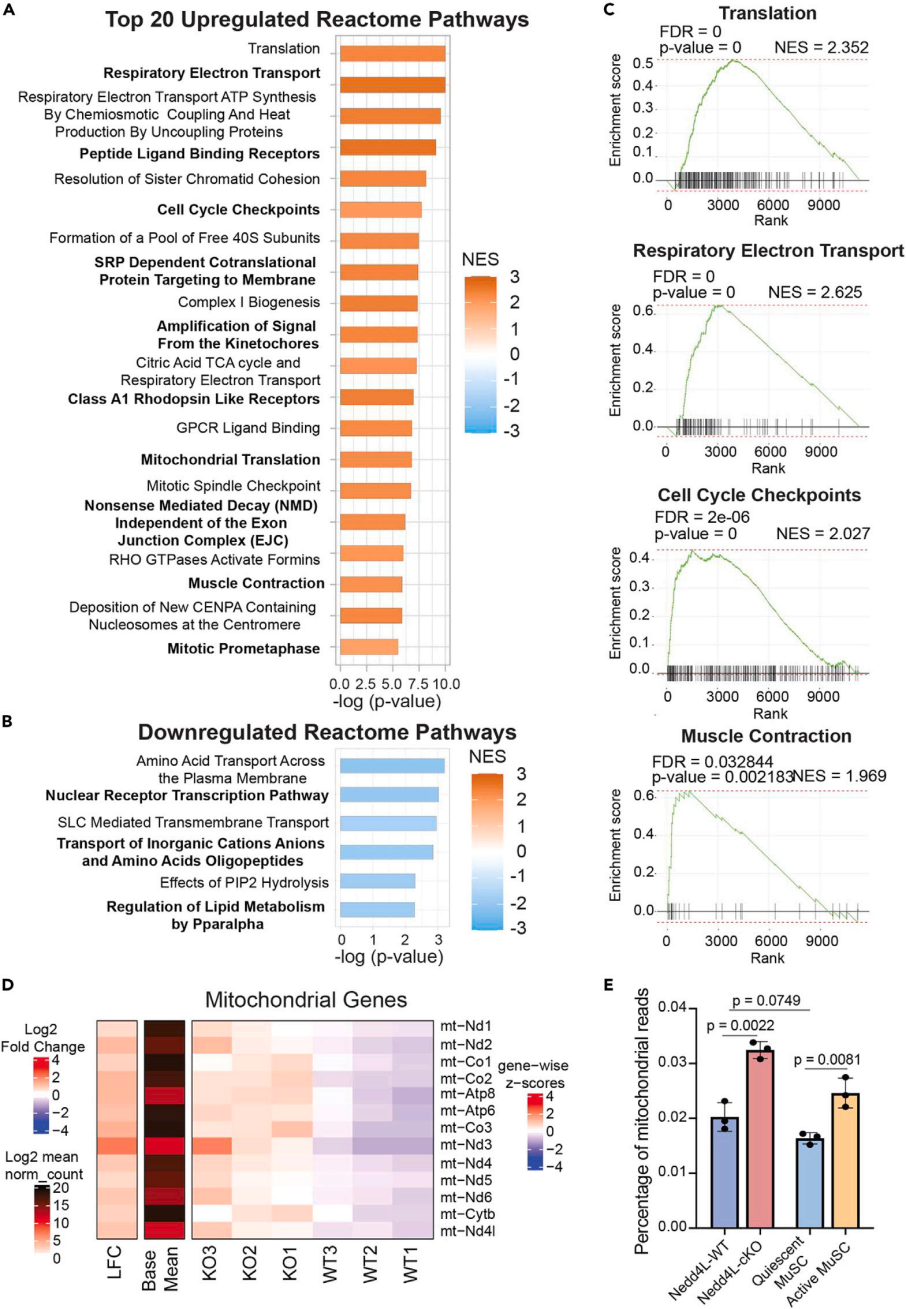
Genetic deletion of Nedd4L upregulates pathways associated with activation and differentiation (A) Top 20 upregulated Reactome pathways in Nedd4L-cKO MuSCs. (B) All downregulated Reactome pathways in Nedd4L-cKO MuSCs. (C) Selected enrichment plots for the translation, respiratory electron transport, cell-cycle checkpoints, and muscle contraction pathways. (D) Heatmap of the expression of the mitochondrial genes in WT and Nedd4L-cKO MuSCs. (E) Bar graph of the percentage of mitochondrial reads from the RNA-seq of WT and Nedd4L-cKO MuSCs, with the quiescent and activated MuSC RNA-seq from publicly available data retrieved from the GEO GSE144871.^14^

When we further analyze the pathways associated with the deregulated genes, we see that the majority of the dysregulated Reactome pathways are upregulated (Figures 3A and 3B). Of the 53 pathways that are deregulated at a threshold of p-adj<0.1, 47 of them are upregulated compared to 6 that are downregulated (Figures 3A and 3B). Interestingly, many of the pathways that are upregulated are associated with MuSC activation, namely translation, respiratory electron transport, cell-cycle checkpoints, and muscle contraction, among others (Figures 3A and 3C).

A similar trend is seen with the Hallmark pathways where we see that the majority of the pathways are upregulated in the Nedd4L-cKO MuSCs (Figure S3A). Likewise, many of the pathways identified are associated with MuSC activation and differentiation. Here, we highlight the myogenesis, G2M checkpoint, and Il6-Jak-Stat3 pathways (Figures S3A and S3B). When we delve further into the genes that are affiliated with these pathways, we identify numerous genes that had previously been reported to be important for late myogenic differentiation, such as *MYMK*, *TNNT3*, and *MYL1* among others, are upregulated in the Nedd4L-cKO MuSCs (Figure S3C). Additionally, a number of cell-cycle-related genes are upregulated in the Nedd4L-cKO condition, for example *CDK1*, *AURKA*, and *BIRC5*, which are known to be downregulated in quiescent stem cells^50^^,^^51^ (Figure S3C). The Jak/Stat pathway has multiple roles in muscle, and its upregulation is associated with both proliferation and differentiation of MuSCs.^52^^,^^53^^,^^54^^,^^55^^,^^56^ We see that *IL6* expression is upregulated in the Nedd4L-cKO MuSCs, along with other inflammatory cytokines (Figure S3C). Interestingly, every mitochondrial gene in the Nedd4L-cKO MuSCs was significantly upregulated, correlating with the upregulation of the oxidative phosphorylation pathway (Figures 3D and S3A). A previous study has shown that there is a metabolic shift in MuSCs during activation and that MuSCs will increase their oxidative phosphorylation.^57^ We also observe an increase in the percentage of mitochondrial reads from the RNA-seq of WT vs Nedd4L-cKO MuSCs, similar to what is seen in the RNA-seq of quiescent and activated MuSCs (Figure 3E). Together, these data indicate that the Nedd4L-cKO MuSCs exhibit a gene signature suggestive of activated MuSCs.

### Nedd4L-cKO MuSCs are transcriptionally similar to active MuSCs

The RNA-seq data suggest that the Nedd4L-cKO MuSCs are no longer in a quiescent state. However, when we compare the expression of the myogenic regulatory factors (MRFs), we see that there is no difference in the expression of any of the MRFs, and only a slight non-significant decrease in *Pax7* expression, possibly due to its reduced mRNA stability in the Nedd4L-cKO (Figures S2D and S4A–S4E). Further, there is no significant increase in expression of the proliferation marker *Ki67* in the Nedd4L-cKO cells (Figure S4F). Together, this would indicate that MuSCs from the Nedd4L-cKO mouse have not fully activated and are not proliferating. Therefore, we posit that the genetic deletion of Nedd4L leads to MuSCs entering a state of G_alert_,^9^ where the cells have not broken quiescence but are closer to activation than a fully quiescent WT MuSC.

**Figure 4:**
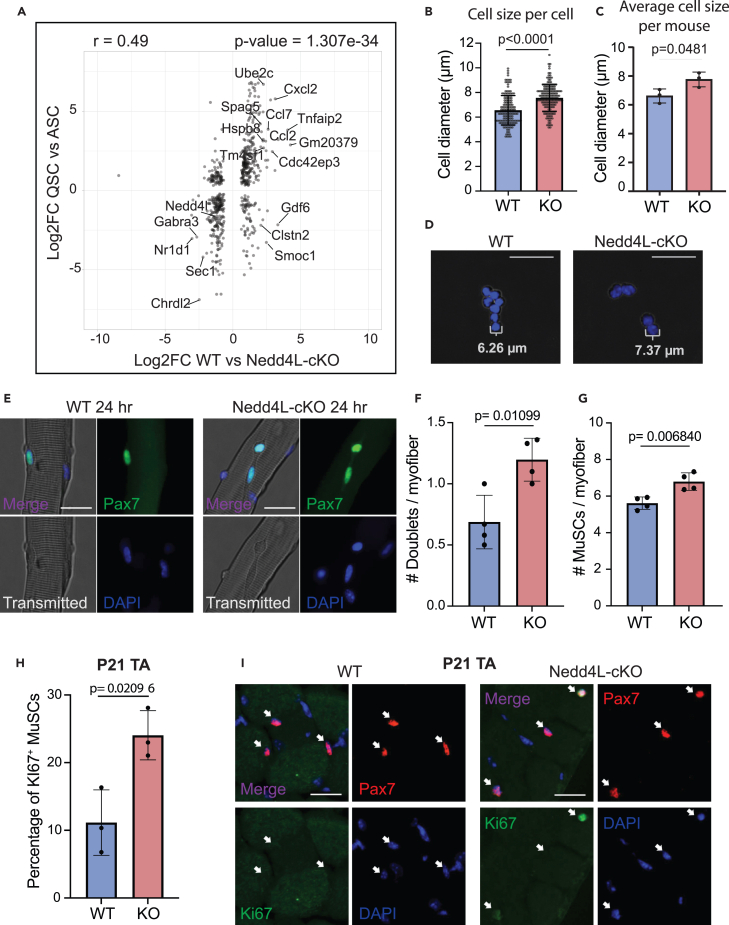
Nedd4L-cKO MuSCs are transcriptionally similar to active MuSCs (A) Scatterplot comparing the LFC of WT vs. Nedd4L-cKO with the LFC of quiescent MuSCs (QSCs) vs. activated muscle stem cells (ASCs). The QSC and ASC sequencing data are publicly available and retrieved from GSE144871.^14^ Genes with a mean expression of over 200 normalized counts and an adjusted *p*-value <0.1 were plotted. Genes with an absolute LFC >2 in both conditions were labeled, with Nedd4L being manually labeled. (B) Bar graph of the cell size of freshly isolated MuSCs from WT and Nedd4L-cKO mice, *n* = 3 mice; 163 WT cells and 184 KO cells; two-tailed t test, data presented as mean ± SD. (C) Bar graph of the cell size of freshly isolated MuSCs from WT and Nedd4L-cKO mice; each point represents the average cell size per mouse, *n* = 3 mice; two-tailed t test, data presented as mean ± SD. (D) Representative images of freshly isolated MuSCs. (E) Representative images of EDL myofibers 24 h post-isolation stained for Pax7. Scale bar: 25 μm. (F) Bar graph of the number of MuSC doublets per myofiber, *n* = 4 mice; two-tailed t test, data presented as mean ± SD. (G) Bar graph of the number of MuSCs per myofiber, *n* = 4 mice; two-tailed t test, data presented as mean ± SD. (H) Bar graph representing the percentage of Ki67^+^ MuSCs from WT and Nedd4L-cKO P21 TA muscle, *n* = 3 mice; two-tailed t test, data presented as mean ± SD. (I) Representative images of TA cross-sections from P21 WT and Nedd4L-cKO mice, stained for Pax7 and Ki67. Scale bar: 25 μm. *n* = 3 male mice; two-tailed t test; data are presented as mean ± SD.

To confirm this, we compared the changes in the transcriptome of WT vs. Nedd4L-cKO MuSCs to the changes in the transcriptome of quiescent (QSC) vs. activated (ASC) MuSCs (Figure 4A). The QSC vs. ASC data were retrieved from publicly available data (GSE144871).^14^ The QSC cells are from uninjured muscle, whereas the ASCs are from the TA and gastrocnemius muscles 3 days post-cardiotoxin (CTX)-induced injury. We see from this comparison that the changes in the transcriptome between WT and Nedd4L-cKO are similar to the changes that occur during injury-induced activation (Figure 4A). Importantly, in the ASC transcriptome we see a downregulation in the expression of Nedd4L (Figure 4A).

The state of G_alert_ is affiliated with an increase in cell size and a shorter time to cell division.^9^ We see in freshly sorted MuSCs that the Nedd4L-cKO condition has significantly larger cells (Figures 4B–4D). Additionally, in EDL myofibers cultured for 24 h, we found that there are significantly more MuSC doublets and a higher number of MuSCs in the Nedd4L-cKO (Figures 4E–4G). This would imply that the Nedd4L-cKO MuSCs more readily divide when cultured compared to their WT counterparts. Further, in the TA cross-sections from P21 (postnatal day 21) mice, we see that the Nedd4L-cKO MuSCs have a higher proportion of those that are Ki67 positive (Figures 4H and 4I). At 3 weeks of age, MuSCs will enter quiescence for the first time.^58^ This higher percentage of Ki67 cells suggests that the genetic deletion of Nedd4L results in a delay in the initial acquisition of quiescence. We then wanted to assess whether the MuSCs of fully grown adult mice were also expressing Ki67 at a higher rate. When we stained the TA cross-sections of 12-month-old mice, we found that there was no difference in the number of MuSCs that were Ki67 positive (Figures S4G–S4I). Therefore, adult MuSCs are not actively cycling. However, when we injured the TA muscle and allowed the muscle to regenerate for 21 days, we found that there was a higher percentage of MuSCs that remained Ki67 positive (Figures S4J and S4K). Therefore, the Nedd4L-cKO MuSCs exhibit a delay in the acquisition of quiescence in the case of regeneration, similar to what is observed at 3 weeks of age.

G_alert_ is characterized by the induction of the mTOR pathway.^9^ To determine whether the Nedd4L-cKO MuSCs have an upregulated mTOR pathway, EDL-associated MuSCs were stained for phospho-S6 kinase at 0 h, 6 h, and 24 h post-isolation (Figures S5A–S5F). We see a significant increase in the phospho-S6 kinase levels in the Nedd4L-cKO condition. To confirm these findings, freshly FACS-sorted MuSCs were also stained for phospho-S6 kinase. Once again, we see that the Nedd4L-cKO MuSCs exhibit a significantly higher level of phospho-S6 kinase (Figures S5G–S5H). We then assessed the percentage of MyoD-expressing MuSCs at 0 h, 6 h, and 24 h post-isolation (Figures S5I–S5N). Similarly, we determined that there was a higher percentage of Nedd4L-cKO MuSCs that expressed MyoD during quiescence and the early stages of activation. Together, the data presented here strengthen the hypothesis that Nedd4L maintains MuSC quiescence and with its genetic deletion the cells enter a state reminiscent of G_alert_.

### Genetic deletion of Nedd4L alters the proteome of freshly isolated MuSCs

**Figure 5:**
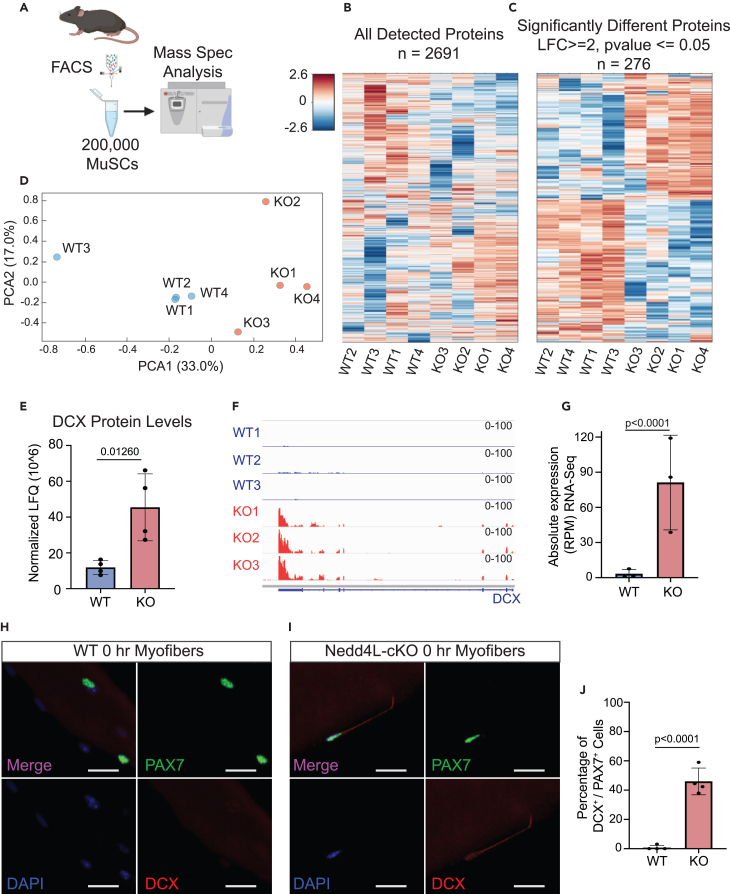
Genetic deletion of Nedd4L alters the proteome of freshly isolated MuSCs (A) Diagram of the design of the mass spectrometry experiment performed on 200,000 freshly isolated WT and Nedd4L-cKO MuSCs, generated with Biorender.com. Mice were pooled to obtain enough MuSCs, *n* = 7 WT, and 10 knockout mice. (B) Heatmap of all detected proteins in 200,000 freshly isolated MuSCs from WT and Nedd4L-cKO mice. (C) Heatmap of the protein levels in WT and Nedd4L-cKO MuSCs for all significantly different proteins (LFC ≥2, *p* value ≤0.05). (D) PCA plot of WT and Nedd4L-cKO MuSCs. (E) Bar graph representing the DCX protein levels from the Mass spec. *n* = 4; two-tailed t test, data presented as mean ± SD. (F) IGV tracks of RNA-seq reads for Dcx. (G) Bar graph of the absolute expression (RPM) of Dcx in WT and Nedd4L-cKO mice. *n* = 4 male mice; two-tailed t test, data presented as mean ± SD. (H) Immunofluorescence for DCX and PAX7 in WT MuSCs associated with freshly isolated (0 h) EDL myofibers. (I) Immunofluorescence for DCX and PAX7 in Nedd4L-cKO MuSCs associated with freshly isolated EDL myofibers. (J) Bar graph representing the percentage of Dcx^+^/Pax7^+^ MuSCs associated to freshly isolated EDL myofibers. *n* = 4; two-tailed t test, data presented as mean ± SD.

Nedd4L is a member of the UPS system and as such regulates the proteome of a cell. To determine how the loss of Nedd4L affects the proteins in quiescent MuSCs, we performed label-free quantification (LFQ) of proteins with nano LC-MS/MS (Figure 5A). Every sample consisted of 200,000 MuSCs, necessitating the pooling of the muscle from multiple mice.

When we analyzed the proteome of freshly isolated WT and Nedd4L-cKO MuSCs, we were able to detect 2,691 proteins and found that 276 of them were significantly different between the conditions (LFC ≥2, *p* value ≤0.05) (Figures 5B and 5C). We observed that this caused a distinct proteomic profile between the WT and Nedd4L-cKO MuSCs as seen by the separate clustering on a PCA plot (Figure 5D). We confirm with the mass spectrometry data the loss of CHRDL2 protein in the Nedd4L-cKO MuSCs (Figure S6A).

A particularly interesting protein that was detected in the Nedd4L-cKO cells is doublecortin (DCX) (Figure 5E). DCX is a 40-kDa protein and is most well-known for its role in neuronal migration and neurogenesis.^59^^,^^60^^,^^61^ This protein has also been shown to have a role in MuSCs; through sequencing it was demonstrated that the gene is only expressed in the cases of neonatal MuSCs, activation, and in the dystrophic (mdx) mouse model.^62^ Further, it was demonstrated to have a key role in the differentiation of MuSCs and their fusion to pre-existing fibers during regeneration.^63^ This led us to compare the transcriptomic expression of *Dcx* from our RNA-seq data and found that the gene is significantly upregulated in the Nedd4L-cKO MuSCs, while having almost no detectable expression in the WT MuSCs (Figures 5F and 5G). To validate the expression of DCX protein in quiescent MuSCs, we stained MuSCs associated to freshly isolated EDL myofibers, for PAX7 and DCX. From this we observed that although almost no WT MuSCs were positive for DCX protein, a large percentage (approximately 45%) of Nedd4L-cKO MuSCs were positive (Figures 5H and 5I). This level of DCX-positive MuSCs was maintained in older mice, aged 6 months (Figures S6B and S6C).

To determine whether DCX is a factor in G_alert_, we examined the contralateral TA muscle from an injured mouse, which is known to induce G_alert_. We stained cross-sections of these contralateral TAs for DCX and Ki67 in the WT and Nedd4L-cKO conditions. Interestingly, we observed that there was an increase in the percentage of DCX-positive MuSCs in the contralateral TAs of the WT mice, although not to the extent of the KO condition (Figures S6D–S6G). Additionally, we determined that there was a higher percentage of Ki67-positive cells in the Nedd4L-cKO condition, possibly indicating that the genetic deletion of Nedd4L permits the MuSCs to enter the cell cycle more readily (Figures S6H and S6I). Together, we posit that the genetic deletion of Nedd4L leads to a change in the proteome, resulting in the MuSCs entering similar to G_alert_ and being primed to differentiate and repair damaged myofibers. Further, the expression of DCX is a marker for this intermediary stage of MuSC activation.

### Chromatin accessibility is unaltered by the genetic deletion of Nedd4L

**Figure 6:**
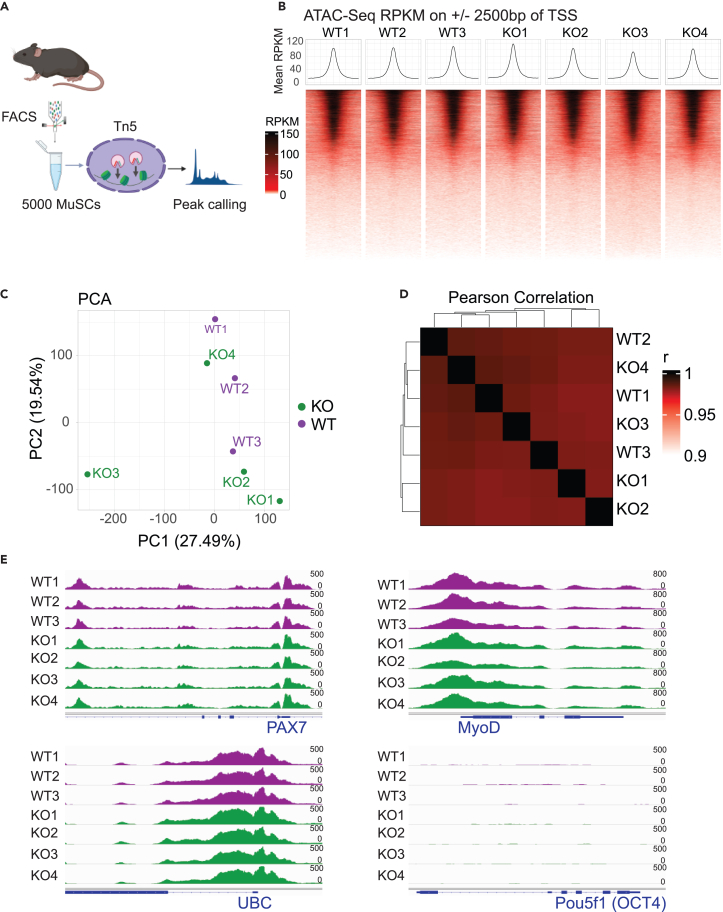
Chromatin accessibility is unaltered by the genetic deletion of Nedd4L (A) Schematic of the ATAC-seq experiment performed on 5,000 freshly isolated MuSCs, generated with Biorender.com. *n* = 3 WT and *n* = 4 Nedd4L-cKO male mice aged 5–6 weeks. (B) Pile-up heatmap of the ±2,500 bp region around TSS of all genes in each ATAC-seq sample. (C) PCA plot of WT and Nedd4L-cKO MuSC ATAC-seq samples. (D) Heatmap of the sample-to-sample Pearson correlation of WT and Nedd4L-cKO MuSC ATAC-seq samples. (E) IGV tracks of ATAC-Seq reads for myogenic genes Pax7 and MyoD, housekeeping gene ubiquitin (UBC), and Pou5f1 (OCT4) as a negative control.

Breaking quiescence and entry into activation is a complex process that results in numerous transcriptomic and biochemical changes in MuSCs, along with an alteration in the chromatin state.^5^^,^^17^ We sought to determine how the chromatin of these cells that were shifted closer to activation was affected. To accomplish this, we performed assay for transposase accessible chromatin with sequencing (ATAC-seq) on freshly isolated WT and Nedd4L-cKO MuSCs (Figure 6A).

Interestingly, we found that there is no difference in chromatin accessibility between the WT and Nedd4L-cKO MuSCs. We verified the quality of our ATAC-seq and found that the results, including the pile-up of reads around TSS and peak annotations, showed a typical profile expected from ATAC-seq (Figure 6B). We see that there is no clustering in the PCA, nor any difference in the Pearson correlation between the samples, regardless of their genotype (Figures 6C and 6D). As a positive control, we visualized PAX7, MYOD1, and the housekeeping gene UBC and saw no differences between samples in the accessibility at the promotor of these genes (Figure 6E). Finally, as a negative control, the promoter of *Pou5f1* (*OCT4*) was inaccessible as expected (Figure 6E). When we looked at individual genes that were identified from our RNA-seq as having a change in their expression, we again see that there is no difference in chromatin accessibility (Figure S7).

Together, these data indicate that the transition from a deeper state of quiescence (PAX7^+^/DCX^−^) to the PAX7^+^/DCX^+^ state does not lead to any changes in chromatin accessibility.

### Nedd4L-cKO MuSCs are more prone to differentiation

**Figure 7:**
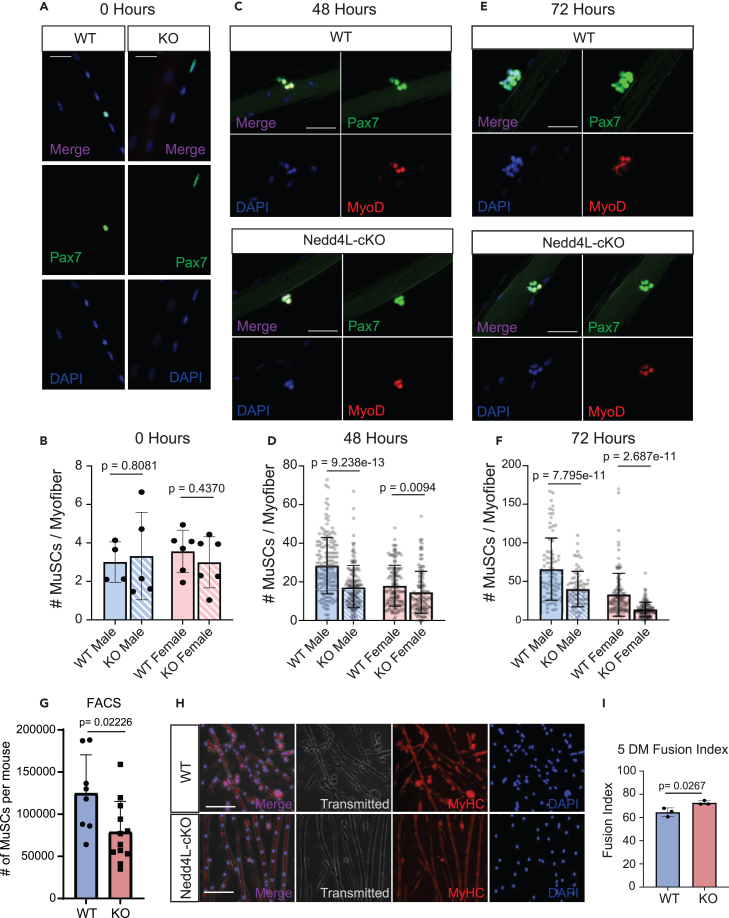
Nedd4L-cKO MuSCs are more prone to differentiation (A) Representative images of isolated EDL myofibers 0 h post-isolation, stained for PAX7. Scale bar: 25 μm. (B) Bar graph representing the number of MuSCs per myofiber 0 h post-isolation in male and female WT and Nedd4L-cKO mice, aged 6–8 weeks. *n* = 4–6 mice; two-tailed t test, data presented as mean ± SD. (C) Representative images of isolated EDL myofibers 48 h post-isolation, stained for PAX7 and MyoD1. Scale bar: 25 μm. (D) Bar graph representing the number of MuSCs per myofiber 48 h post-isolation in male and female WT and Nedd4L-cKO mice, aged 6–8 weeks. *n* = 131–162 myofibers from 4 to 6 mice; two-tailed t test, data presented as mean ± SD. (E) Representative images of isolated EDL myofibers 72 h post-isolation, stained for PAX7 and MyoD1. Scale bar: 25 μm. (F) Bar graph representing the number of MuSCs per myofiber 72 h post-isolation, in male and female WT and Nedd4L-cKO mice, aged 6–8 weeks. *n* = 107–117 myofibers from 4 to 6 mice; two-tailed t test, data presented as mean ± SD. (G) Bar graph of the total number of MuSCs isolated from the hindlimb muscles by FACS. *n* = 8 male WT mice and 12 male Nedd4L-cKO mice, aged 6–8 weeks; two-tailed t test, data presented as mean ± SD. (H) Representative images of WT and Nedd4L-cKO primary myotubes grown in differentiation media for 5 days and stained for myosin heavy chain (MyHC). (I) Bar graph representing the fusion index of WT and Nedd4L-cKO grown for 5 days in differentiation media. *n* = 3; two-tailed t test, data presented as mean ± SD.

To determine the functional effect of the genetic deletion of Nedd4L in MuSCs, EDL myofibers were isolated from 6- to 8-week-old male and female mice and cultured for 0, 24, 48, and 72 h. When stained for PAX7, we found that there was no difference in the number of MuSCs in freshly isolated myofibers from Nedd4L-cKO and WT mice (Figures 7A and 7B). However, as they grew in culture for 48 and 72 h, it was observed that the Nedd4L-cKO MuSCs could not expand as rapidly as the WT, as seen by the reduced number of PAX7^+^ cells in the Nedd4L-cKO condition (Figures 7C–7F). Additionally, when we quantify the number of MuSCs in the hindlimb muscles of WT and Nedd4L-cKO mice, we see that there are significantly less MuSCs in the Nedd4L-cKO mice (Figure 7G). It is possible that the numbers from one individual muscle would show no difference in MuSC numbers, but when analyzing the entire hindlimb muscles we are able to detect a decrease in MuSCs. To validate that this loss of MuSCs was not due to apoptosis, myofibers that were cultured for 72 h were stained for cleaved caspase-3, a marker of apoptosis (Figures S8A and S8B). We found no difference in the percentage of MuSCs that were apoptotic between the WT and Nedd4L-cKO.

The inability of the Nedd4L-cKO MuSCs to properly expand may be due to defects in proliferation. To determine this, we performed an EdU assay on cultured WT and KO primary myoblasts for 12 and 24 h. We see that there is no difference in the number of EdU-positive cells between the conditions, thereby indicating that there is no difference in proliferation upon genetic deletion of Nedd4L (Figures S8C and S8D).

An increase in the level of differentiation could also explain the lower number of MuSCs. When performing a differentiation assay for 5 days, we observed that the fusion index in the Nedd4L-cKO myotubes was significantly higher than in the WT, indicating an increase in differentiation (Figures 7H and 7I). As previously mentioned from our RNA-seq data, there is also large number of genes involved in late differentiation that are upregulated in the Nedd4L-cKO MuSCs. Together, these data suggest that the loss of Nedd4L pushes the MuSCs toward precocious differentiation, thereby hindering the expansion of the MuSC pool. However, in cultured primary myoblasts, we see no difference in the protein level of MyoD or Pax7, nor do we see any difference in the protein level of myogenin in 5DM cultured myotubes (Figures S8E and S8F). Interestingly, although we do not see a change in MuSC numbers in EDL myofibers at T0 in young mice, we do see a decrease in the EDLs of 12-month-old Nedd4L-cKO mice compared to WTs of the same age (Figures S8G and S8H). This could be indicative of a gradual decline in MuSC number due to precocious differentiation as the mice age. To further assess the differentiation potential of the Nedd4L-cKO MuSCs, we analyzed the percentage of EDL-associated MuSCs expressing Pax7, MyoD, and myogenin at 48 and 72 h (Figures S9A–S9H). From these data, we found that there was no difference in the percentage of MuSCs expressing these myogenic factors.

In the TA of young mice, we also do not observe a difference in the MuSC pool (Figures S10A–S10D). However, in older mice there is a decrease in the number of MuSCs (Figures S10E and S10F), similar to what is seen in the EDL muscle. To assess the effect the genetic deletion of Nedd4L has on the ability of the muscle to regenerate, we performed CTX injury on the TA of both young and old mice. Interestingly, there was no observable difference between the WT and Nedd4L-cKO MuSCs’ ability to regenerate injured muscle. Both conditions were able to expand their MuSC pool in response to the injury and regenerate the muscle (Figures S10C–S10F).

## Discussion

Accumulating evidence suggest that adult stem cell quiescence is a rather dynamic state, with the cells continuously monitoring and responding to a plethora of signals by modulating its depth of quiescence.^5^^,^^50^^,^^64^^,^^65^ Quiescence may therefore be viewed as a gradient, rather than an on/off switch, with the cells able to move from a state of deep quiescence to one viewed as G_alert_, passing through several checkpoints. G_alert_ may itself possess a gradient ranging from more quiescent to more active.

Previous studies have shown the importance of proteostasis for MuSC function and quiescence.^28^^,^^66^^,^^67^ In this study, we show that Pax7 directly contributes to MuSC quiescence via its transcriptional control of the E3 ubiquitin ligase Nedd4L.

When Nedd4L is genetically deleted in MuSCs *in vivo*, there is an alteration in the transcriptome with an upregulation of genes involved in MuSC activation and differentiation. Notably, we observed the loss of expression of *Nr1d1*, *Calcr*, and *Chrdl2* in the Nedd4L-cKO MuSCs, all of which have been shown to play a role in the maintenance of MuSC quiescence.^45^^,^^46^^,^^47^^,^^48^^,^^49^ Interestingly, although there is an upregulation of genes involved in myogenic differentiation, most of them are characteristic of late differentiation. Of note, *Mymk* is upregulated in the Nedd4L-cKO MuSCs, which is essential for the fusion of myoblasts, a process that is important for the late stages of differentiation.^68^^,^^69^ Despite these transcriptomic changes, none of the MRFs are deregulated, nor do we observe an increase in *Ki67* expression in the Nedd4L-cKO MuSCs compared to their WT counterpart. This led us to conclude that the loss of Nedd4L results in the cells entering a state similar to G_alert_, as they are less quiescent than their WT counterparts. Nedd4l-deficient MuSCs exhibit a larger cell size, upregulate mitochondrial genes, an increase in MyoD and phospho-S6 kinase but they have not yet re-entered the cell cycle.

An interesting finding in this study is the revelation that despite the numerous transcriptomic changes seen in the Nedd4L-cKO MuSCs there is no corresponding alteration in chromatin accessibility, as seen by the ATAC-seq performed on freshly isolated MuSCs (Figure 6). This may be indicative that the genes responsible for early activation are in regions of bivalent chromatin. A previous study shows that a large number of genes in the quiescent MuSC are bivalent^17^ and that bivalency allows for a gene to be transcriptionally repressed, while retaining the ability to quickly respond to external cues and be upregulated.^70^ This phenomenon would be consistent with the needs of a quiescent MuSC to maintain its quiescence while still being able to respond rapidly to the cues to activate in case of injury.

Furthermore, our data point to a new marker for the intermediary state between quiescence and activation. PAX7^+^/DCX^+^/Ki67^−^, as we see that only the Nedd4L-cKO MuSCs would consistently express DCX. Further, we see that MuSCs that are in G_alert_ have an increase in the expression of the DCX protein. Previous work on this protein has shown that, in MuSCs, DCX is only expressed in the *in vivo* context, seen only during injury, in neonatal mice, or in mdx mice but not in cultured myoblasts.^62^^,^^71^ A previous study by Ogawa et al. demonstrated that DCX is involved in the differentiation of MuSCs, specifically with regard to the fusion of MuSCs to pre-existing myofibers, in a manner that does not follow the classic myogenic differentiation pathway.^71^ They characterize the DCX-positive cells identified in their study as being “non-classical myoblasts” that differentiate without the detected expression of MyoD or myogenin. This finding could explain why there is a large number of late differentiation genes that are upregulated in the Nedd4L-cKO cells, with few genes associated with early stage differentiation exhibiting any significant change in their expression, along with our observation that there is no difference in the percentage of MuSCs expressing MyoD or myogenin 48 h and 72 h post-isolation. Further, this push toward differentiation and fusion with existing fibers may explain the difficulty that the Nedd4L-cKO MuSCs have in expanding when the myofibers are cultured. This is despite not having any observed defect in proliferation, nor an increase in the apoptosis as indicated by cleaved caspase-3 staining.

An important role of Nedd4L is that of a tumor suppressor; it is most often downregulated in cancer cells.^29^^,^^35^^,^^36^^,^^37^^,^^38^ This tumor suppressor role of Nedd4l may explain its function in the maintenance of MuSC quiescence. We hypothesize that in quiescent MuSCs, Nedd4L would act as a tumor suppressor and inhibit the MuSCs ability to enter the cell cycle.

Our results suggest that Nedd4L may be maintaining quiescence by degrading proteins via the UPS. One possibility is that MuSCs continuously produce proteins necessary for their activation, while simultaneously degrading them. The rationale for this phenomenon is that injury is completely unpredictable, both in timing and in intensity. MuSCs must therefore always be primed to enter the cell cycle in order to rapidly respond to these unexpected injuries and regenerate the tissue as effectively as possible, as shown by recent experiments.^28^ Therefore, active recycling of protein involved in early stages of MuSCs activation can be viewed as a cell survival premium to ensure against the unpredictable nature of tissue injury. Our study indicates that regulation of Nedd4l by PAX7 maintains MuSCs in a quiescent state, which are poised for rapid re-entry into the cell cycle. Further research will need to be conducted to fully explore the role of the UPS in MuSC function and on the role protein turnover plays in stem cell quiescence.

### Limitations of the study

A limitation of this study is the challenge in working with quiescent MuSCs in their *in vivo* context. This study explored the role of the E3 ubiquitin ligase Nedd4L, an enzyme that ubiquitinates proteins for their targeted degradation. Current methods and technologies are limited in their utility for robust investigation of the protein content and dynamics of a quiescent MuSC *in vivo*. Beyond being a very rare cell type, the MuSC cannot be removed from its *in vivo* niche nor manipulated without compromising its quiescence state. To quantify the proteins in quiescent MuSCs, we performed mass spectrometry. Although this approach provides an overview of the proteome, it alone cannot identify targets of an E3 ligase. Techniques such as TurboID can be used to identify potential targets; however, the use of this tool on quiescent MuSCs is also challenging. These techniques could be performed on myoblasts; however, the information gained would be of limited value due to being performed on a different cell type that is not in a quiescent state. From our mass spectrometry data, we chose to highlight both DCX, a known marker of activation and differentiation, as well as CHRDL2, a marker of quiescence, to illustrate how the Nedd4L-cKO MuSCs are less quiescent than their WT counterparts. However, we would not make the claim that either of these proteins are targets of Nedd4L. It is also important to note that although our data indicate a change in the proteome of the Nedd4L-cKO MuSCs, it is possible that the role of Nedd4L in quiescent MuSCs is mediated not only by regulating protein stability through the UPS but also by protein trafficking, another function of ubiquitination. Additionally, the genetic mouse model used in this study results in the genetic deletion of Nedd4L in MuSCs and all of their progenitors, including the fully differentiated myofibers. It is possible that the knockout of Nedd4L in the myofiber could also alter their secretome and thereby affect the communication between myofibers and their associated MuSCs.

## STAR★Methods

### Key resources table

**Table:** 

REAGENT or RESOURCE	SOURCE	IDENTIFIER
**Antibodies**		

PAX7	DSHB	RRID: AB_528428
MYOD1 (G1)	Santa Cruz	Cat# Sc-377460; RRID: AB_2813894
Myogenin	DSHB	RRID: AB_2146602
MF20	DSHB	RRID: AB_2147781
GAPDH	Cell Signaling Technology	Cat# 97166; RRID: AB_2756824
KI67	Abcam	Cat# AB15580; RRID: AB_443209
NEDD4L	Proteintech	Cat# 13690-1-AP; RRID: AB_2149326
DCX	Cell Signaling Technology	Cat# 4604; RRID: AB_561007
CHRDL2	Invitrogen	Cat# MA5-24056; RRID: AB_2606392
Phospho-P70 S6 Kinase	Millipore Sigma	Cat# MABS82; RRID: AB_11204517
Anti-mouse ITGA7-Alexa647	R&D systems	Cat# FAB3518R; RRID: AB_10973483
Anti-mouse PE-CD45	Invitrogen	Cat# 12-0451-82; RRID: AB_465668
Anti-mouse PE-CD11b	Invitrogen	Cat# 12-0112-82; RRID: AB_2734869
Anti-mouse PE-CD31	BD Pharmigen	Cat# 553373; RRID: AB_394819
Anti-mouse PE-Sca1/Ly6A-E	BD Pharmigen	Cat# 553108; RRID: AB_394629
Alexa Fluor 568 goat anti-mouse IgG1	Invitrogen	A21124
Alexa Fluor 488 goat anti-rabbit IgG	Invitrogen	A11008
Alexa Fluor 594 goat anti-mouse IgG2b	Invitrogen	A21145
Alexa Fluor 594 goat anti-rabbit IgG	Invitrogen	A11012
Alexa Fluor 488 goat anti-mouse IgG1	Invitrogen	A21121
Alexa Fluor 488 goat anti-rat IgG	Invitrogen	A11006

**Chemicals, peptides, and recombinant proteins**		

Fetal Bovine Serum	Wisent	080-450
10% Goat Serum	Thermofisher	50062Z
Horse Serum	Wisent	065-150
BSA	Sigma Aldrich	9048-46-8
Glycine	Bioshop	GLN001.5
Sucrose	Millipore Sigma	S7903
Triton-X_100_	BioShop	TRX777.500
Ham’s F10 Nutrient Mix	Thermofisher	11550043
DMEM	Thermofisher	11965118
PBS 1X	Wisent	311-013-CL
Penicillin-Streptomycin (10,000 U/mL)	Thermofisher	15140122
bFGF	Thermofisher	PHG0266
Collagen from rat tail tendon	Millipore Sigma	11179179001
2-Methylbutane	Millipore Sigma	270342
Frozen Section Compound	VWR	95057-838
Collagenase D	Millipore Sigma	1108888201
Dispase II	Millipore Sigma	4942078001
Hoechst	Thermofisher	62249
Paraformaldehyde	Electron microscopy sciences	15714
Prolong Gold Antifade Mountant with DNA Stain DAPI	Thermofisher	P36931
Ampure XP beads	Beckman Coulter	A63882
Cardiotoxin	Latoxan	L8102-1MG

**Critical commercial assays**		

SMART-Seq HT Kit	Takara Bio	634456
Nextera XT DNA Library Preparation Kit	Illumina	FC-131-1024
Nextera XT Index Kit	Illumina	FC-131-1001
Click-iT Edu Alexa Fluor 555 Imaging kit	Invitrogen	C10338

**Deposited data**		

RNA-Seq of MuSCs and Myoblasts	This study	GEO: GSE230622
ATAC-Seq of MuSCs	This study	GEO: GSE230622
RNA-Seq of Myofibers	Blackburn et al. 2019	GEO: GSE138591
Pax7 ChIP-Seq	Lilja et al. 2017	GEO: GSE89977
RNA-Seq of Quiescent and Activated MuSCs	Brun et al. 2022	GEO: GSE144871

**Experimental models: Organisms/strains**		

C57BL/6J	Jackson Laboratory	000664
Nedd4l^tm1.1Hkb^	Hiroshi Kawabe Laboratory	N/A
Pax7-Cre Pax7tm1(cre)Mrc/J	Jackson Laboratory	010530
Nedd4L^fl/fl^ Pax7^cre/+^	This manuscript	N/A

**Software and algorithms**		

Adobe Illustrator	Adobe	https://www.adobe.com/ca/
Prism	Graphpad	https://www.graphpad.com/

### Resource availability

#### Lead contact

Further information and requests for resources and reagents should be directed to and will be fulfilled by the lead contact, Dr. Vahab Soleimani (vahab.soleimani@mcgill.ca).

#### Materials availability

Materials and reagents are available upon request.

#### Data and code availability

• RNA-Seq and ATAC-Seq data have been deposited in the GEO public data repository. GEO accession numbers of the data that is used in this study are listed in the key resources table.
• No original code was produced in this study.
• Any additional information required to reanalyze the data reported in this paper is available from the lead contact upon request.

### Experimental model and study participant details

All experiments with mice were performed in accordance with the Canadian Council on Animal Care (CCAC) guidelines for proper animal care. The protocol was approved by the McGill University Animal Care Committee (UACC) (Protocol #7152). All animal procedures in this study for mice were approved and performed in accordance with the guidelines of the McGill University Animal Care Committee (UACC). The mouse line used in this study is Nedd4L^fl/fl^ Pax7^cre/+^ with a C57BL/6 background. Experiments were performed on both male and female mice, between the ages of 1 – 12 months. In all experiments, mice are age and sex matched. *In vitro* experiments were performed on primary myoblasts derived from male mice.

### Method details

#### Intramuscular injection of MG132 into the tibialis anterior

8-week-old C57BL/6 mice were injected intramuscularly with 50 μL of 10mM MG132 dissolved in DMSO, once every 24 hours for 3 days. Control mice were injected with 50 μL of DMSO for the same time period. After the final injection, TAs were collected 24 hours later and fixed in 0.5% PFA for 2 hours at 4°C. The TAs were placed in 20% sucrose at 4°C overnight and then frozen in frozen section compound using liquid nitrogen cooled 2-methylbutane.

#### Fluorescence activated cell sorting of muscle stem cells

The hindlimb muscles were dissected and minced using scissors and subsequently digested in 5 mL of FACS digestion media (HAM’s F10 with 2.4 U/mL Collagenase D, 10 U/mL Dispase II, and 0.5 mM CaCl_2_) for 30 minutes at 37°C in a 5% CO_2_ tissue culture incubator. The muscle was pulsed in a centrifuge and the supernatant was transferred to 8 mL of FBS on ice. The remaining muscle pellet is triturated with a pipette and 5 mL of FACS digestion media is added and the muscle was incubated for another 30 minutes. The digested muscle was transferred to the same FBS. Sample was then filtered through a 40 μm cell strainer. The cells are pelleted by centrifugation at 500 G for 18 minutes at 4°C. The cell pellet was resuspended in 500 μL of 2% FBS and 0.5mM EDTA in PBS and were incubated for 20 minutes at room temperature with the following antibodies: Alexa647-conjugated to anti-mouse ITGA7, PE-CD31, PE-CD45, PE-CD11b, PE-SCA1 and Hoechst 33342. After the cells were washed with 10 mL of 2% FBS in PBS and centrifuged at 500 G for 15 minutes at 4°C. Cells were resuspended in 800 μL of 2% FBS in PBS and filtered through a 40 μm cell strainer. The ITGA7^+^/Hoechst^+^/CD31^−^/CD45^−^/CD11b^−^/SCA1^−^ population was sorted with a FACS aria fusion, as previously described.^72^

#### Cell culture

Muscle stem cells that were isolated by FACS were cultured on collagen coated plates in growth media (Ham’s F10 supplemented with 20% FBS, 1% penicillin/streptomycin and 5 ng/mL of bFGF) in a cell incubator set to 37°C and 5% CO_2_. Cells were passaged upon attaining 70% confluence.

#### Creating PAX7 or NEDD4L over-expressing primary myoblasts

Stable cell lines of primary myoblasts over-expressing PAX7 or NEDD4L were generated as previously described.^73^ Briefly, retrovirus particles containing the plasmids of interest were generated in Phoenix helper-free retrovirus producer lines with lipofectamine. The viral supernatant was collected 48 h after transfection and subsequently applied to cultured primary myoblasts for 8 h. Afterwards the cells were washed twice with 1 × PBS and grown for 48 h in normal growth media. Puromycin selection media (HAM's F10 supplemented with 20% FBS, 5 ng/ml bFGF, 1% penicillin/streptomycin, and 2.5 μg/ml puromycin) was then given to the cells for 1 week. Finally, the cells were maintained in low puromycin maintenance media (HAM's F10 supplemented with 20% FBS, 5 ng/ml bFGF, 1% penicillin/streptomycin, and 1.25 μg/ml puromycin).

#### EdU incorporation assay

Cultured primary myoblasts were treated with EdU as described in the Click-It Plus EdU Alexa Fluor 555 Imaging Kit (Invitrogen C10638). Briefly, growth media with 10 μM of EdU was added to primary myoblasts seeded on collagen coated chamber slides. The cells were then cultured for 12 or 24 hours. Media was removed and the cells washed twice with PBS and then fixed with 3.2% paraformaldehyde. EdU staining was performed as described in the kit.

#### Myoblast differentiation

Cultured myoblasts were grown to 90% confluence and differentiated in DMEM supplemented with 5% horse serum (HS) and 1% penicillin/streptomycin for 5 days. After 3 days the media was replaced with fresh differentiation media for the duration of the assay.

#### Preparation of SMART RNA-Seq libraries from freshly sorted muscle stem cells

1000 MuSCs were FACS sorted directly into 10 μL SMART lysis buffer (1 μL SMART-Seq Reaction Buffer (95% SMART-Seq 10X lysis buffer, 5% RNAse Inhibitor), 9 μL ddH_2_O) in a 0.2 mL microtube. 1 μL of 3’ SMART-Seq CDS primer II A was added, and the samples were incubated for 3 minutes at 72°C, then placed on ice. 12.5 μL of the template switching master mix (0.7 μL of nuclease free water, 8 μL of one-step buffer, 1 μL SMART-Seq HT oligonucleotide, 0.5 μL RNase inhibitors, 0.3 μL of SeqAMP DNA polymerase and 2 μL of SMARTscribe reverse transcriptase) was added to each sample. The cDNA was amplified for 11 cycles as described previously.^42^^,^^74^

The cDNA was purified using AMPure beads at a 1:1 volume/volume ratio. The beads were washed twice with 200 μL of 80% ethanol. The cDNA was eluted from the beads with 17 μL of TE buffer (10 mM Tris-HCl, 1mM EDTA, pH 8.0). 250 pg of cDNA in a final volume of 1.25 μL, was transferred to a fresh microtube. With the Nextera XT DNA Library Preparation Kit, 2.5 μL of TD buffer and 1.25 μL of ATM was added to the sample and incubated for 5 minutes at 55°C. The samples were removed from the heat and had 1.25 μL of NT buffer added, the samples were then incubated at RT for 5 minutes. For the amplification of the libraries and the incorporation of unique indices, 1.25 μL each of an i7 and an i5 index was added as well as 3.75 μL of NPM PCR master mix were added. The libraries were amplified for 12 cycles in a thermocycler using the conditions previously described.^42^^,^^74^

The libraries were purified and size selected using AMPure beads at a 0.85X volume ratio. The AMPure beads were washed twice with 200 μL of 80% ethanol and the libraries were eluted with 20 μL of resuspension buffer. The libraries were sequenced with a NextSeq500 75bp single end reads.

#### Preparation of primary myoblast RNA-Seq libraries

MuSCs were FACS sorted as in the “Fluorescence activated cell sorting of muscle stem cells” section described above and cultured *in vitro* for 3 days in growth media (Ham’s F10, 20% FBS, 1% penicillin/streptomycin, 5 ng/mL bFGF). After 3 days, the cells were trypsinized with 0.125% trypsin and the cells were pelleted at 1500 RPM for 5 minutes. The cells were resuspended in PBS and counted. 1000 cells were transferred to SMART lysis buffer (1 μL SMART-Seq Reaction Buffer (95% SMART-Seq 10X lysis buffer, 5% RNAse Inhibitor), 9 μL ddH_2_0) at a final volume of 12.5 μL. Myoblast RNA-seq libraries were then generated in the same manner as the MuSCs, described above.

#### RNA-seq analysis

To determine the differential gene expression, mRNA expression was quantified with Kallisto v0.46.1 (parameters: --single --fragment-length 200 --sd 20).^75^ The GENCODE v.M28 basic gene annotations was used for transcriptome information.^76^ Transcript-level abundance estimates were collapse to the gene level using the “tximport”.^77^ Differentially expressed genes were identify using DESeq2.^78^ P values were adjusted for multiple testing using the Independent Hypothesis Weighting procedure.^79^

Reads were aligned with HISAT2 and converted to bigwigs using bamCoverage (version 3.5.1). Read counts were normalized as RPM.^80^^,^^81^

#### Analysis of differential mRNA stability

Differential mRNA stability was inferred as previously described.^82^ Briefly, exonic and intronic regions from the RNA-seq reads were mapped using annotations acquired from Ensembl GRCm39 version 87. The decoupling of the transcriptional and post-transcriptional effects was achieved with DiffRAC determining changes in mRNA stability.

#### Extraction and digestion of proteins for total proteomic analyses

Proteins from FACS sorted mouse muscle stem cell populations were extracted in lysis buffer containing 5% sodium dodecyl sulfate (SDS), 100 mM TRIS pH 7.8. Samples were subsequently heated to 99°C for 10 minutes. The lysate was clarified by centrifugation 14,000 x g for 5 minutes. An aliquot corresponding to 10% of the total volume of lysate was diluted to <1% SDS and used for estimation of protein concentration by bicinchoninic acid assay (BCA). In the remaining sample, protein disulfide bonds were reduced by the addition of tris(2-carboxyethyl)phosphine (TCEP) to a final concentration of 20 mM and incubated at 60°C for 30 minutes. Free cysteines were alkylated using iodoacetamide at a final concentration of 30 mM and subsequent incubation at 37°C for 30 minutes in the dark. An equivalent of 10 μg of total protein was used for proteolytic digestion using suspension trapping (STRAP). In brief, proteins were acidified through the addition of phosphoric acid to a final concentration of 1.3% v/v. The sample was subsequently diluted 6-fold in STRAP loading buffer (9:1 methanol: water in 100 mM TRIS, pH 7.8) and loaded onto an S-TRAP Micro cartridge (Protifi LLC, Huntington NY) and spun at 4000 x g for 2 minutes. Samples were washed three times using 150 μL of STRAP loading buffer. Proteins were then proteolytically digested using trypsin at a 1:10 enzyme to substrate ratio for 2 hours at 47°C. Peptides were sequentially eluted in 50 mM ammonium bicarbonate, 0.1% formic acid in water, and 50% acetonitrile. Peptide containing samples were then desalted using self-made R3-STAGE tips. Desalted peptides were vacuum concentrated and reconstituted in 0.1% trifluoroacetic acid (TFA) prior to analysis by LC-MS/MS.

#### LC-MS/MS acquisition and data analysis

Peptide containing samples were analyzed by data dependent acquisition (DDA) using an Easy-nLC 1200 online coupled to a Q Exactive Plus. Samples were loaded onto the precolumn (Acclaim PepMap 100 C18, 3 μm particle size, 75 μm inner diameter x 2 cm length) in 0.1% formic acid (buffer A). Peptides were separated using a 100-min binary gradient ranging from 3-40% of buffer B (84% acetonitrile, 0.1% formic acid) on the main column (Acclaim PepMap 100 C18, 2 μm particle size, 75 μm inner diameter x 25 cm length) at a flow rate of 300 nL/min. Full MS scans were acquired from m/z 350-1,500 at a resolution of 70,000, with an automatic gain control (AGC) target of 1 x 10^6^ ions and a maximum injection time of 50 ms. The 15 most intense ions (charge states +2 to +4) were isolated with a window of m/z 1.2, an AGC target of 2 x 10^4^ and a maximum injection time of 64 ms and fragmented using a normalized higher-energy collisional dissociation (HCD) energy of 28. MS/MS were acquired at a resolution of 17,500 and the dynamic exclusion was set to 40 s. DDA MS raw data was processed with Proteome Discoverer 2.5 (Thermo Scientific) and searched using Sequest HT against a mouse UniProt FASTA database. The enzyme specificity was set to trypsin with a maximum of 2 missed cleavages. Carbamidomethylation of cysteine was set as fixed modification and oxidation of methionine as variable modification. The precursor ion mass tolerance was set to 10 ppm, and the product ion mass tolerance was set to 0.02 Da. Percolator was used to assess posterior error probabilities and the data was filtered using a false discovery rate (FDR) <1% on peptide and protein level. The Minora feature detector node of Proteome Discoverer was used for label free quantitation (LFQ) based on precursor areas. LFQ abundances were scaled (normalized) based on the total peptide amount per sample. Only proteins quantified with at least 1 protein unique peptide were retained in the data summary. In order to estimate the relative amount of a particular protein within the sample we calculated and ranked proteins based on their normalized spectral abundance factor (NSAF) values (https://doi.org/10.1021/pr060161n). Hierarchical clustering was conducted using the normalized protein LFQ abundances as inputs for Instant Clue (http://www.instantclue.uni-koeln.de/).

#### Preparation of muscle stem cell ATAC-Seq libraries

The ATAC-seq libraries were generated following the OMNI ATAC-seq protocol.^83^ 5000 MuSCs from hindlimbs were sorted using FACS into 30 μL of lysis buffer (10 mM Tris-HCl pH 7.5, 10 mM NaCl, 3 mM MgCl_2_, 0.1% Tween-20, 0.1% NP-40, 0.01% Digitonin) and incubated on ice for 5 minutes and then at room temperature for 3 min. Cells were then washed with 100 μL of wash buffer (10 mM Tris-HCl pH 7.5, 10 mM NaCl, 3 mM MgCl_2_, 0.1% Tween-20) and centrifuged at 800 g for 10 min. 10 μL transposition mix (5 μL of Tagment DNA (TD) buffer, 3.2 μL PBS, 0.89 μL Tn5, 0.1% Tween-20, 0.01% Digitonin and 0.75 μL water) was used to resuspend the cell pellet. The samples were incubated for 20 min at 37°C and were mixed every 5-7 min. DNA purification was then performed with column purification as described (Qiagen, QIAquick PCR Purification Kit Cat: 28104), eluted into a final volume of 20 μL.

Indices were incorporated by adding 30 μL of the PCR reaction mix (10 μL Q5 buffer, 10 μL Q5 enhancer, 1 μL dNTPs, 2.5 μL i7 index, 2.5 μL i5 index, 3.5 μL nuclease free water, 0.5 μL Q5 High Fidelity DNA polymerase). PCR amplification was performed using the previously described conditions for 12 cycles.^84^^,^^85^ The libraries were then size selected and purified with Ampure XP beads at a 1:0.85 (v:v) ratio. After the validation of the libraries with a bioanalyzer, samples were sequenced with NovaSeq6000 Sprime, Paired End (PE) 150bp.

#### Analysis of ATAC-Seq data

Adapters were removed from raw ATAC-seq reads using Trimmomatic (version 0.39, parameters: LEADING:10 TRAILING:10 MINLEN:50).^86^ Paired reads were aligned to mm10 using BWA-MEM (version 0.7.17).^87^ Samtools was used to index and filter (version 1.16.1, mapping quality > 30).^88^^,^^89^ Duplicated reads were removed using Picard (version 2.26.3).^90^

ATAC-seq peaks were called with MACS2 (version 2.2.7.1, parameters: --nomodel --shift -100 --extsize 200 –broad, --gsize mm).^91^^,^^92^

#### Isolation and culture of EDL myofibers

The skin from the hindlimb was removed and the TA muscle was dissected out to provide access to the EDL. The EDL was dissected by cutting the distal and proximal tendon. The EDL was placed in a 1.5 mL tube containing 800 μL of digestion buffer (1000 U/mL collagenase in unsupplemented DMEM) for 1 hour at 37°C and 5% CO_2_. Using a large bore glass pipette, coated with 10% HS in DMEM, the EDL was transferred to a 6-well plate with 2 mL of unsupplemented DMEM in each well. The wells had previously been coated with 10% HS in DMEM for 30 minutes. The EDL muscle was gently triturated with a coated large bore glass pipette to disassociate the myofibers. The myofibers were then either fixed immediately for the 0 hour time point, or allowed to rest in the unsupplemented DMEM for 1 hour in a cell incubator, set to 37°C and 5% CO_2_, before having the DMEM replaced with growth media (DMEM supplemented with 15% FBS, 1% chick embryo extract, 1% penicillin/streptomycin, 5 ng/mL bFGF).

#### Immunofluorescence of EDL myofibers

Isolated EDL myofibers were transferred using a small-bore glass pipette, coated with 10% HS in DMEM, to a 24-well plate. The myofibers were fixed with 400 μL of 4% PFA in PBS for 5 minutes at room temperature. The myofibers were washed three times in 400 μL of 0.1% Triton-X in PBS. Blocking and permeabilization were performed in tandem using 400 μL of blocking buffer (1% Triton-X, 10% goat serum, 6% horse serum, 2% BSA, and 0.1 M of glycine in PBS) for 1 hour at RT. Myofibers were incubated with primary antibodies diluted in blocking buffer overnight at 4°C. The next day, the samples were washed three times in 400 μL of 0.1% Triton-X in PBS. Next, the samples were incubated with secondary antibody diluted in blocking buffer for 1 hour at RT. The myofibers were then washed three times in 400 μL of 0.1% Triton-X in PBS and afterwards transferred to a glass slide with mounting solution containing DAPI.

#### Cardiotoxin injury of the tibialis anterior muscle

Mice were anesthetized with isoflurane and the site of the injection was wiped with 70% ethanol. The tibialis anterior was injected with 50 μL of 10mM of cardiotoxin. The muscle was permitted to regenerate for 21 days before being collected for cross sections and immunofluorescence analysis.

#### Immunofluorescence of tibialis anterior muscle cross sections

The tibialis anterior muscle (TA) was dissected and immediately fixed in 1 mL of 0.5% paraformaldehyde (PFA) in PBS at 4°C for 2 hours. The PFA was then replaced with 1 mL of 20% sucrose in dH_2_O and incubated overnight at 4°C. The TA was then frozen in clear sectioning liquid with liquid chilled isopentane.

The cross sections were cut at a width of 9 μm with a cryostat at -25°C. The cross sections were placed on a microscope slide and a hydrophobic barrier was drawn around them using a PAP pen. The cross sections were permeabilized for 15 minutes at RT with 0.5% Triton-X in PBS. The sections were then washed 3 times with PBS and then were incubated for 2 hours at RT in blocking buffer (10% goat serum, 3% BSA, 0.1M glycine). The sections were incubated with the primary antibodies diluted in blocking buffer overnight at 4°C in a humid chamber. Then the sections were washed 3 times with 0.1% Triton-X in PBS. The cross sections were incubated with the secondary antibodies diluted 1:400 in blocking buffer for 1 hour at RT. The cross sections were then washed 3 times with 0.1% Triton-X in PBS. Lastly, the slides were mounted with Prolong gold antifade mounting solution with DAPI and the cover slips were sealed with clear nail polish.

### Quantification and statistical analysis

Statistical analyses were performed using Prism9 (Graphpad). All data is presented as mean ± SD. When two independent groups were compared an unpaired two-tailed t-tests was used. When multiple groups were compared, analysis was done using a 2 way ANOVA. Exact p values are found within the relevant figure panel and a p value of < 0.05 was considered to be significant. Sample sizes for each experiment is described in the corresponding figure legend.
