# Supplementary material for: The E3 ubiquitin ligase Nedd4L preserves skeletal muscle stem cell quiescence by inhibiting their activation
Source: iScience. 2024 Jun 11;27(7):110241. doi: 10.1016/j.isci.2024.110241 (PMC11250905; doi:10.1016/j.isci.2024.110241)
Supplement: Document S1. Figures S1–S10 [file mmc1.pdf]

iScience, Volume *27*

Supplemental information

The E3 ubiquitin ligase Nedd4L preserves skeletal muscle stem cell quiescence by inhibiting their activation

Darren M. Blackburn, Korin Sahinyan, Aldo Hernández-Corchado, Felicia Lazure, Vincent Richard, Laura Raco, Gabrielle Perron, René P. Zahedi, Christoph H. Borchers, Christoph Lepper, Hiroshi Kawabe, Arezu Jahani-Asl, Hamed S. Najafabadi, and Vahab D. Soleimani

Figure S1

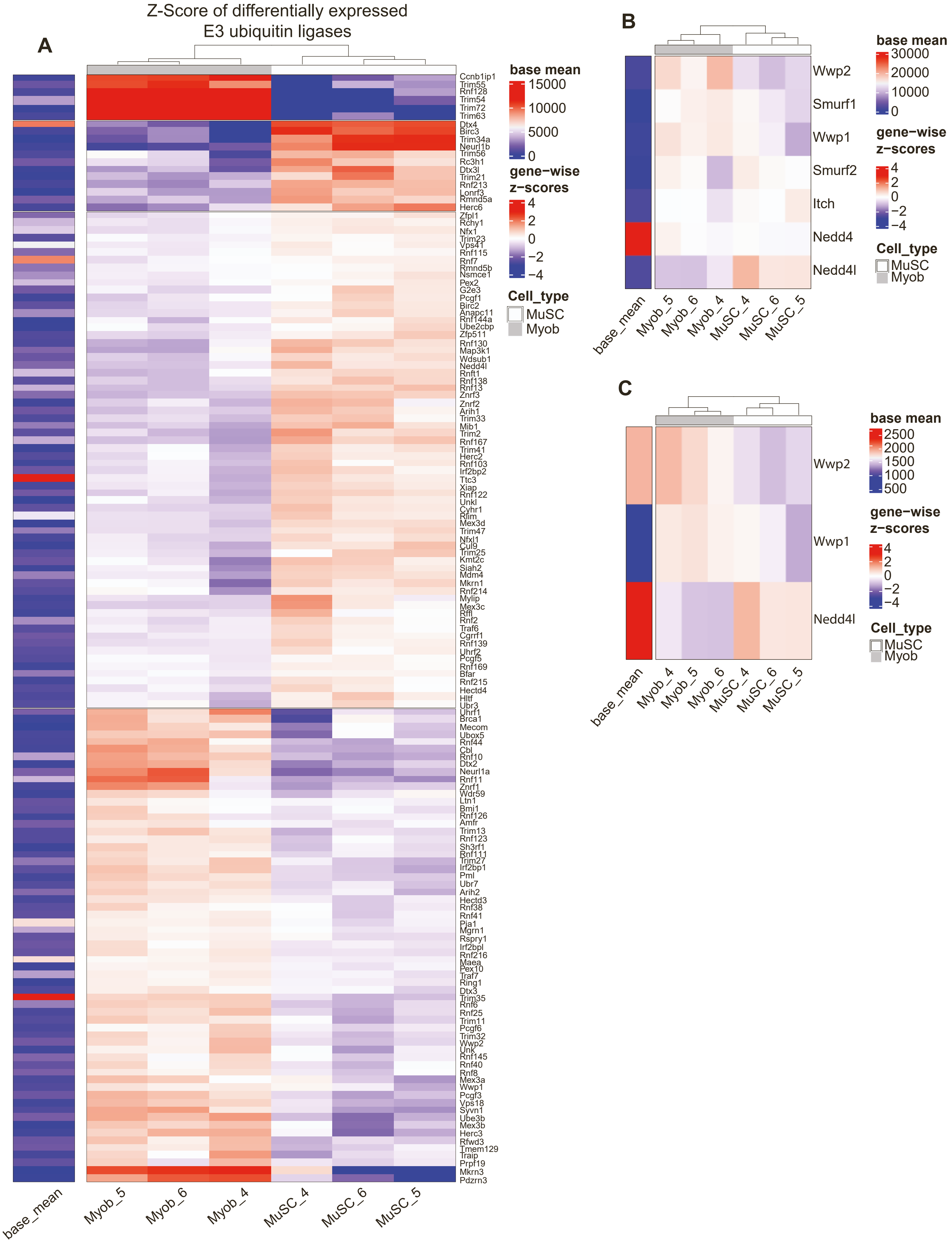

A
Z-Score of differentially expressed
E3 ubiquitin ligases
Ccnb1ip1
Trim55
Rnf128
Trim54
Trim72
Trim63
Dtx4
Birc3
Trim34a
Neurl1b
Trim56
Rc3h1
Dtx3l
Trim21
Rnf213
Lonrf3
Rmnd5a
Herc6
Zfpl1
Rchy1
Nfx1
Trim23
Vps41
Rnf115
Rnf7
Rmnd5b
Nsmce1
Pex2
G2e3
Pcgf1
Birc2
Anapc11
Rnf144a
Ube2cbp
Zfp511
Rnf130
Map3k1
Wdsub1
Nedd4l
Rnft1
Rnf138
Rnf13
Znrf3
Znrf2
Arih1
Trim33
Mib1
Trim2
Rnf167
Trim41
Herc2
Rnf103
Irf2bp2
Ttc3
Xiap
Rnf122
Unkl
Cyhr1
Rlim
Mex3d
Trim47
Nfxl1
Cul9
Trim25
Kmt2c
Siah2
Mdm4
Mkrn1
Rnf214
Mylip
Mex3c
Rffl
Rnf2
Traf6
Cgrrf1
Rnf139
Uhrf2
Pcgf5
Rnf169
Bfar
Rnf215
Hectd4
Hltf
Ubr3
Uhrf1
Brca1
Mecom
Ubox5
Rnf44
Cbl
Rnf10
Dtx2
Neurl1a
Rnf11
Znrf1
Wdr59
Ltn1
Bmi1
Rnf126
Amfr
Trim13
Rnf123
Sh3rf1
Rnf111
Trim27
Irf2bp1
Pml
Ubr7
Arih2
Hectd3
Rnf38
Rnf41
Pja1
Mgrn1
Rspry1
Irf2bpl
Rnf216
Maea
Pex10
Traf7
Ring1
Dtx3
Trim35
Rnf6
Rnf25
Trim11
Pcgf6
Trim32
Wwp2
Unk
Rnf145
Rnf40
Rnf8
Mex3a
Wwp1
Pcgf3
Vps18
Syvn1
Ube3b
Mex3b
Herc3
Rfwd3
Tmem129
Traip
Prpf19
Mkrn3
Pdzrn3
base_mean
Myob_5
Myob_6
Myob_4
MuSC_4
MuSC_6
MuSC_5
base mean
15000
10000
5000
0
gene−wise
z−scores
4
2
0
−2
−4
Cell_type
MuSC
Myob
B
Wwp2
Smurf1
Wwp1
Smurf2
Itch
Nedd4
Nedd4l
base_mean
Myob_5
Myob_6
Myob_4
MuSC_4
MuSC_6
MuSC_5
base mean
30000
20000
10000
0
gene−wise
z−scores
4
2
0
−2
−4
Cell_type
MuSC
Myob
C
Wwp2
Wwp1
Nedd4l
base_mean
Myob_4
Myob_5
Myob_6
MuSC_4
MuSC_6
MuSC_5
base mean
2500
2000
1500
1000
500
gene−wise
z−scores
4
2
0
−2
−4
Cell_type
MuSC
Myob

Figure S2

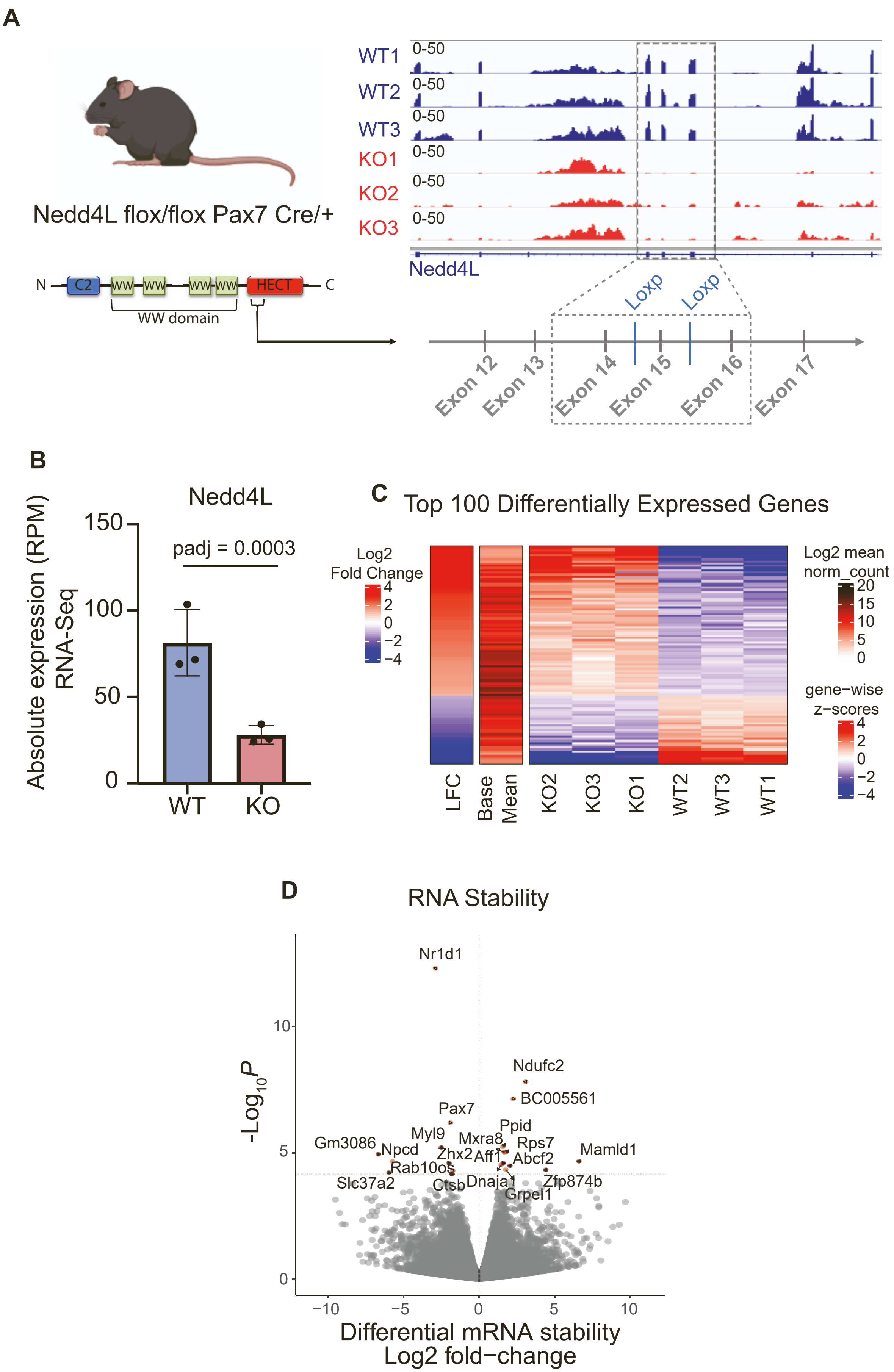

A
Nedd4L flox/flox Pax7 Cre/+
N
C2
WW
WW
WW
WW
HECT
C
WW domain
WT1
WT2
WT3
KO1
KO2
KO3
0-50
Nedd4L
Loxp
Loxp
Exon 12
Exon 13
Exon 14
Exon 15
Exon 16
Exon 17
B
Nedd4L
padj = 0.0003
Absolute expression (RPM) RNA-Seq
150
100
50
0
WT
KO
C
Top 100 Differentially Expressed Genes
Log2 Fold Change
4
2
0
−2
−4
Log2 mean norm_count
20
15
10
5
0
gene−wise z−scores
4
2
0
−2
−4
LFC
Base Mean
KO2
KO3
KO1
WT2
WT3
WT1
D
RNA Stability
Nr1d1
Ndufc2
BC005561
Pax7
Ppid
Myl9
Mxra8
Rps7
Gm3086
Npcd
Zhx2
Aff1
Mamld1
Abcf2
Rab10os
Slc37a2
Ctsb
Dnaja1
Zfp874b
Grpel1
$-\log_{10}P$
10
5
0
−10
−5
0
5
10
Differential mRNA stability
Log2 fold−change

Figure S3

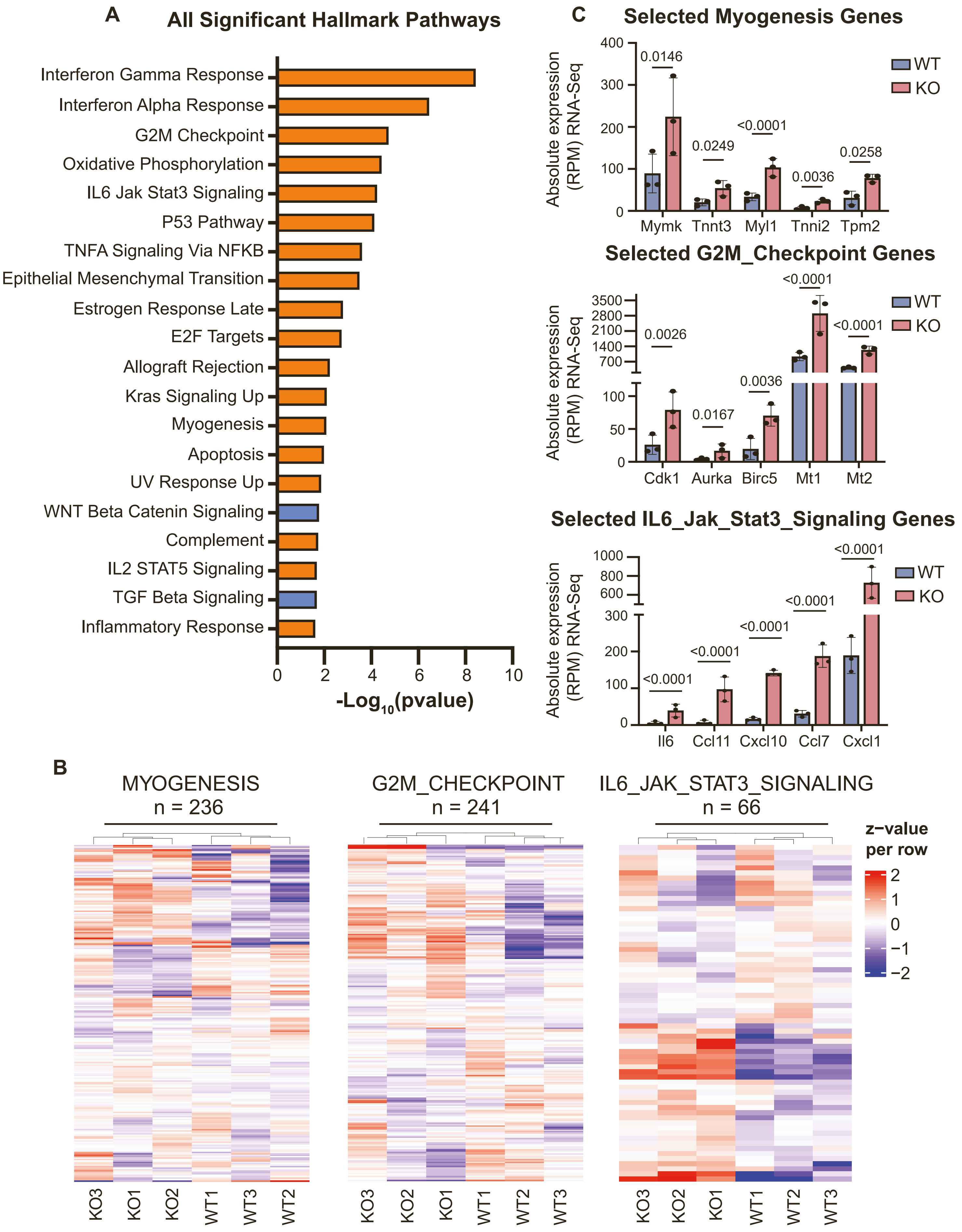
A
All Significant Hallmark Pathways
Interferon Gamma Response
Interferon Alpha Response
G2M Checkpoint
Oxidative Phosphorylation
IL6 Jak Stat3 Signaling
P53 Pathway
TNFA Signaling Via NFKB
Epithelial Mesenchymal Transition
Estrogen Response Late
E2F Targets
Allograft Rejection
Kras Signaling Up
Myogenesis
Apoptosis
UV Response Up
WNT Beta Catenin Signaling
Complement
IL2 STAT5 Signaling
TGF Beta Signaling
Inflammatory Response
0 2 4 6 8 10
-Log10(pvalue)
C
Selected Myogenesis Genes
Absolute expression (RPM) RNA-Seq
0.0146
0.0249
<0.0001
0.0036
0.0258
Mymk Tnnt3 Myl1 Tnni2 Tpm2
WT
KO
Selected G2M_Checkpoint Genes
0.0026
0.0167
0.0036
<0.0001
<0.0001
Cdk1 Aurka Birc5 Mt1 Mt2
Selected IL6_Jak_Stat3_Signaling Genes
<0.0001
<0.0001
<0.0001
<0.0001
<0.0001
Il6 Ccl11 Cxcl10 Ccl7 Cxcl1
B
MYOGENESIS
n = 236
G2M_CHECKPOINT
n = 241
IL6_JAK_STAT3_SIGNALING
n = 66
z−value per row
KO3 KO1 KO2 WT1 WT3 WT2
KO3 KO2 KO1 WT1 WT2 WT3
KO3 KO2 KO1 WT1 WT2 WT3

Figure S4

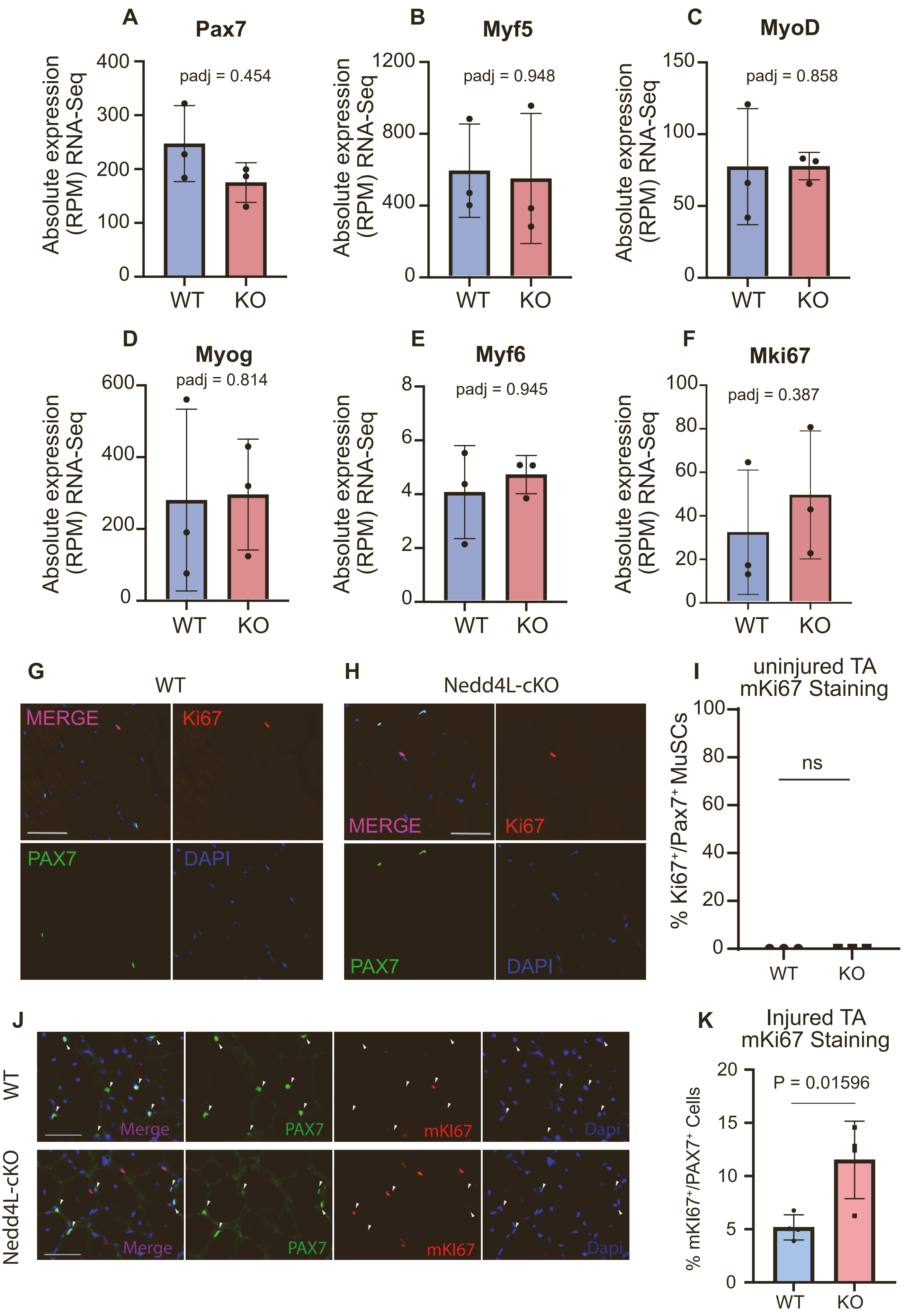
A
Pax7
padj = 0.454
Absolute expression (RPM) RNA-Seq
400
300
200
100
0
WT
KO
B
Myf5
padj = 0.948
1200
800
400
0
WT
KO
C
MyoD
padj = 0.858
150
100
50
0
WT
KO
D
Myog
padj = 0.814
600
400
200
0
E
Myf6
padj = 0.945
8
6
4
2
0
F
Mki67
padj = 0.387
100
80
60
40
20
0
G
WT
MERGE
Ki67
PAX7
DAPI
H
Nedd4L-cKO
I
uninjured TA mKi67 Staining
% Ki67+/Pax7+ MuSCs
ns
J
Nedd4L-cKO
Merge
mKI67
Dapi
K
Injured TA mKi67 Staining
% mKI67+/PAX7+ Cells
P = 0.01596
20
15
10
5
0

Figure S5

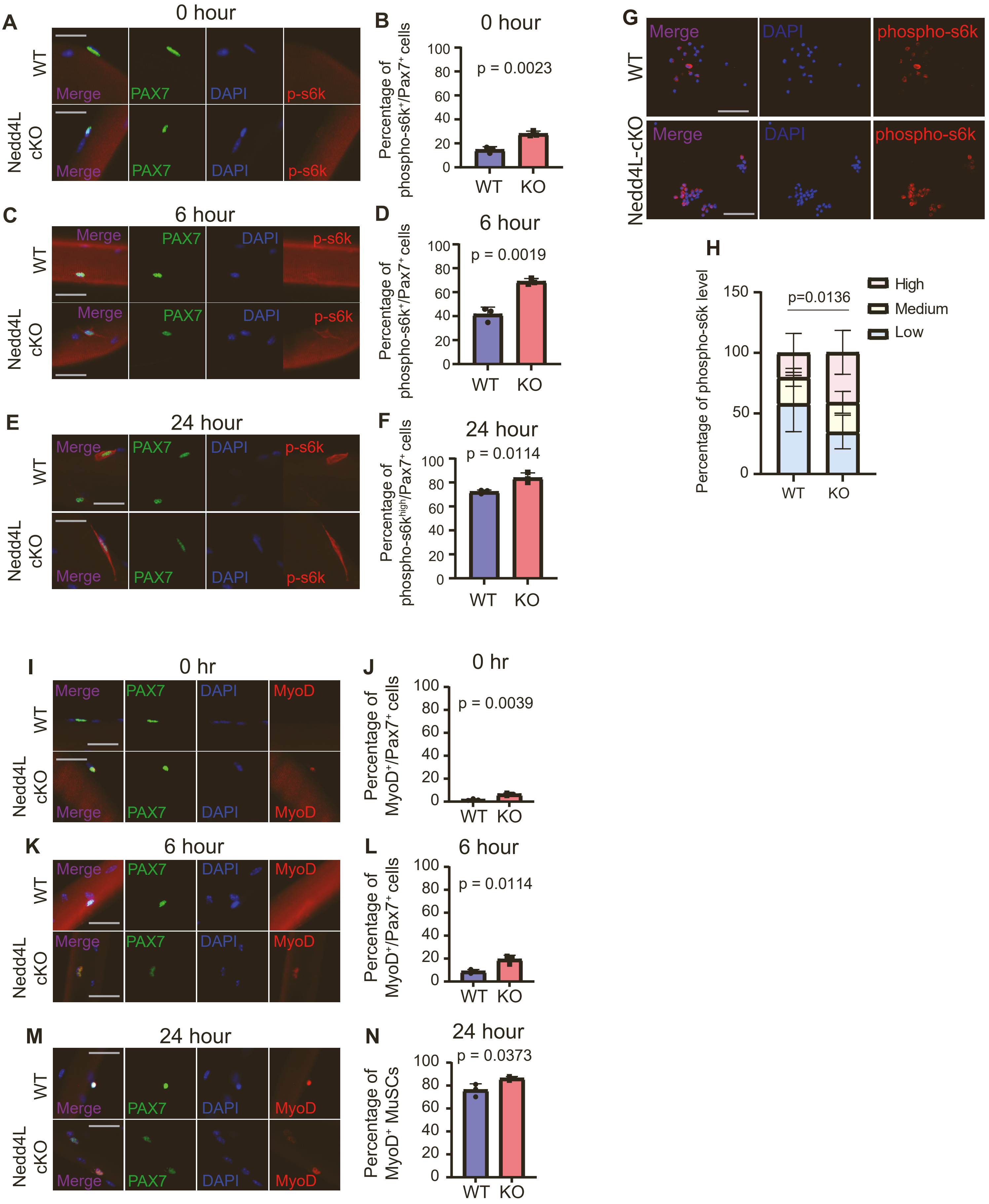

A
0 hour
WT
Nedd4L cKO
Merge
PAX7
DAPI
p-s6k
B
0 hour
Percentage of phospho-s6k+/Pax7+ cells
p = 0.0023
WT
KO
C
6 hour
D
6 hour
p = 0.0019
E
24 hour
F
24 hour
Percentage of phospho-s6khigh/Pax7+ cells
p = 0.0114
G
Merge
DAPI
phospho-s6k
WT
Nedd4L-cKO
H
Percentage of phospho-s6k level
p=0.0136
High
Medium
Low
I
0 hr
MyoD
J
0 hr
Percentage of MyoD+/Pax7+ cells
p = 0.0039
K
6 hour
L
6 hour
p = 0.0114
M
24 hour
N
24 hour
Percentage of MyoD+ MuSCs
p = 0.0373

Figure S6

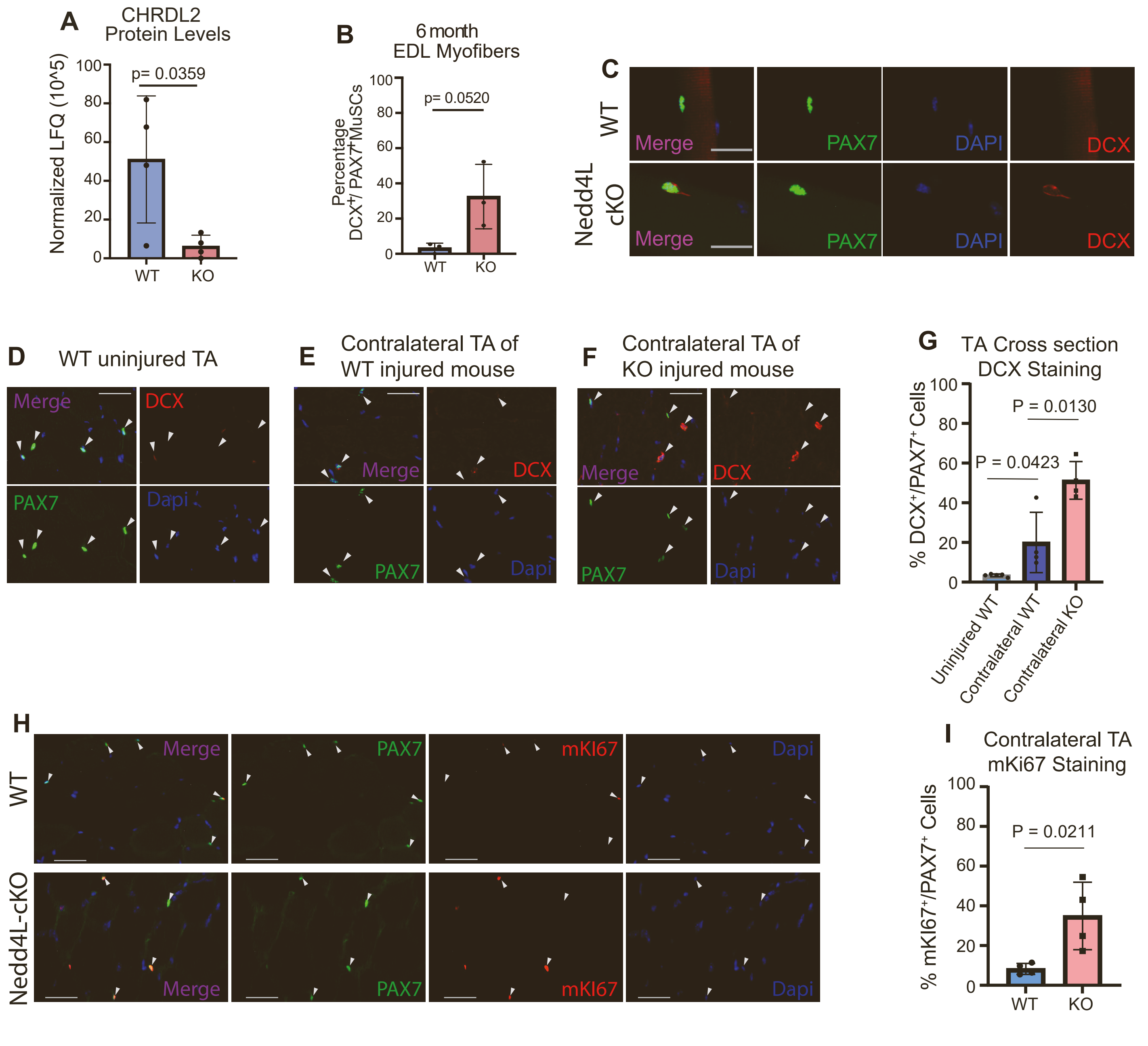
A
CHRDL2 Protein Levels
Normalized LFQ (10^5)
p= 0.0359
WT
KO
B
6 month EDL Myofibers
Percentage DCX+/ PAX7+MuSCs
p= 0.0520
WT
KO
C
WT
Nedd4L cKO
Merge
PAX7
DAPI
DCX
D
WT uninjured TA
Merge
DCX
PAX7
Dapi
E
Contralateral TA of WT injured mouse
Merge
DCX
PAX7
Dapi
F
Contralateral TA of KO injured mouse
Merge
DCX
PAX7
Dapi
G
TA Cross section DCX Staining
% DCX+/PAX7+ Cells
P = 0.0130
P = 0.0423
Uninjured WT
Contralateral WT
Contralateral KO
H
WT
Nedd4L-cKO
Merge
PAX7
mKI67
Dapi
I
Contralateral TA mKi67 Staining
% mKI67+/PAX7+ Cells
P = 0.0211
WT
KO

Figure S7

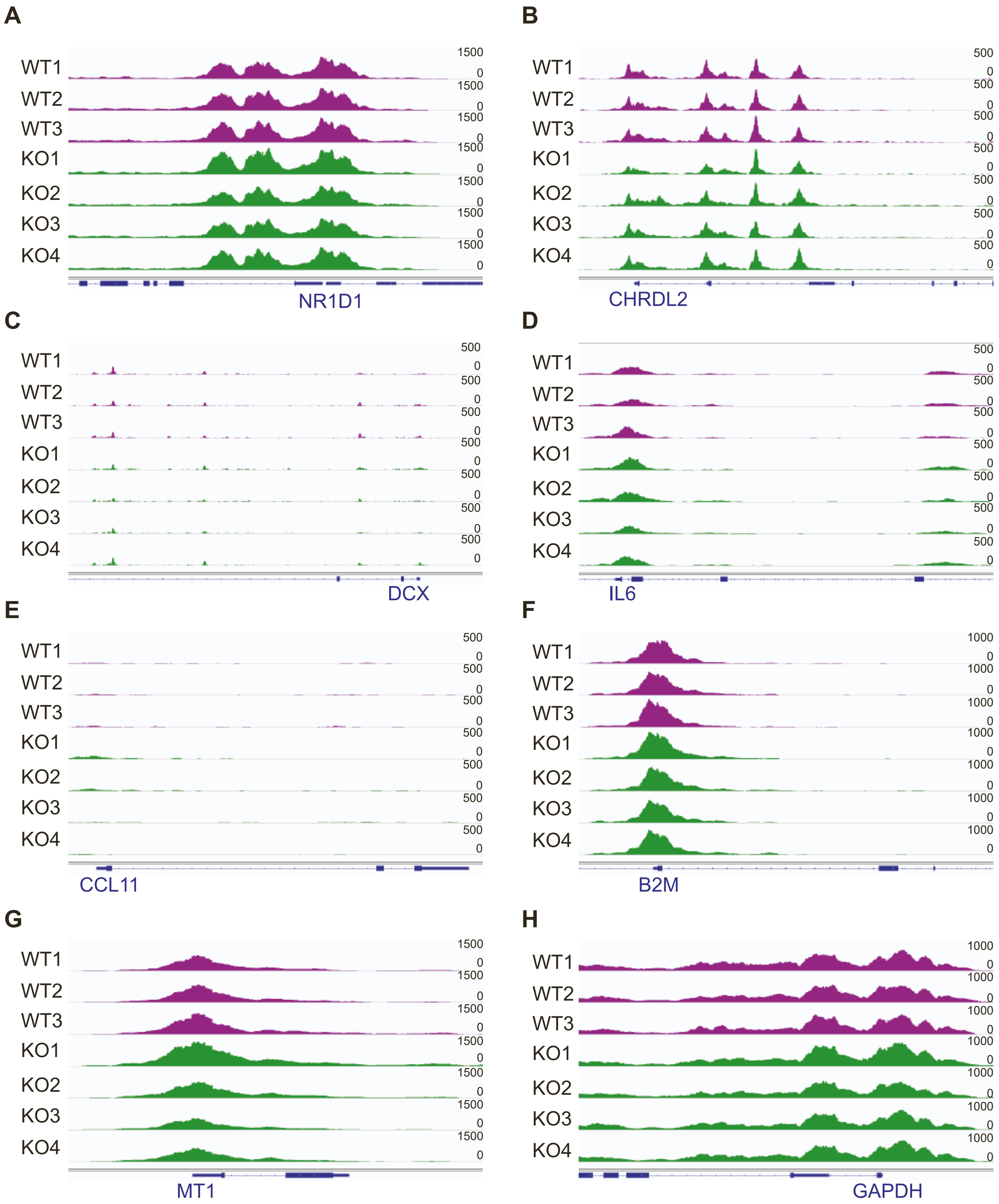
A
WT1
WT2
WT3
KO1
KO2
KO3
KO4
1500
0
NR1D1
B
500
0
CHRDL2
C
500
0
DCX
D
IL6
E
CCL11
F
1000
B2M
G
MT1
H
GAPDH

Figure S8

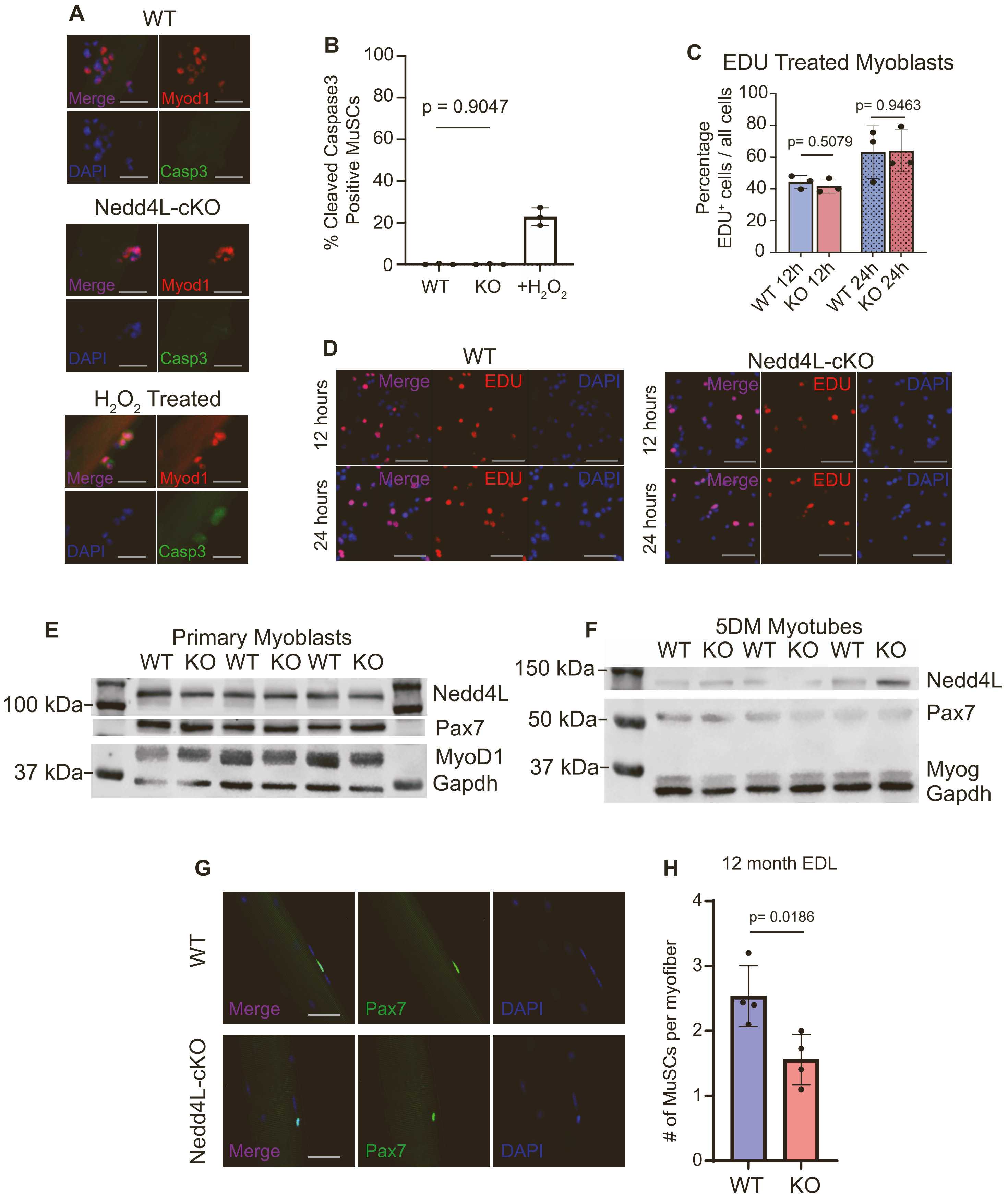
A
WT
Merge
Myod1
DAPI
Casp3
Nedd4L-cKO
Merge
Myod1
DAPI
Casp3
H2O2 Treated
Merge
Myod1
DAPI
Casp3
B
% Cleaved Caspase3 Positive MuSCs
100
80
60
40
20
0
p = 0.9047
WT
KO
+H2O2
C
EDU Treated Myoblasts
Percentage EDU+ cells / all cells
100
80
60
40
20
0
p= 0.5079
p= 0.9463
WT 12h
KO 12h
WT 24h
KO 24h
D
WT
Nedd4L-cKO
12 hours
24 hours
Merge
EDU
DAPI
E
Primary Myoblasts
WT KO WT KO WT KO
100 kDa
37 kDa
Nedd4L
Pax7
MyoD1
Gapdh
F
5DM Myotubes
WT KO WT KO WT KO
150 kDa
50 kDa
37 kDa
Nedd4L
Pax7
Myog
Gapdh
G
WT
Nedd4L-cKO
Merge
Pax7
DAPI
H
12 month EDL
# of MuSCs per myofiber
4
3
2
1
0
p= 0.0186
WT
KO

Figure S9

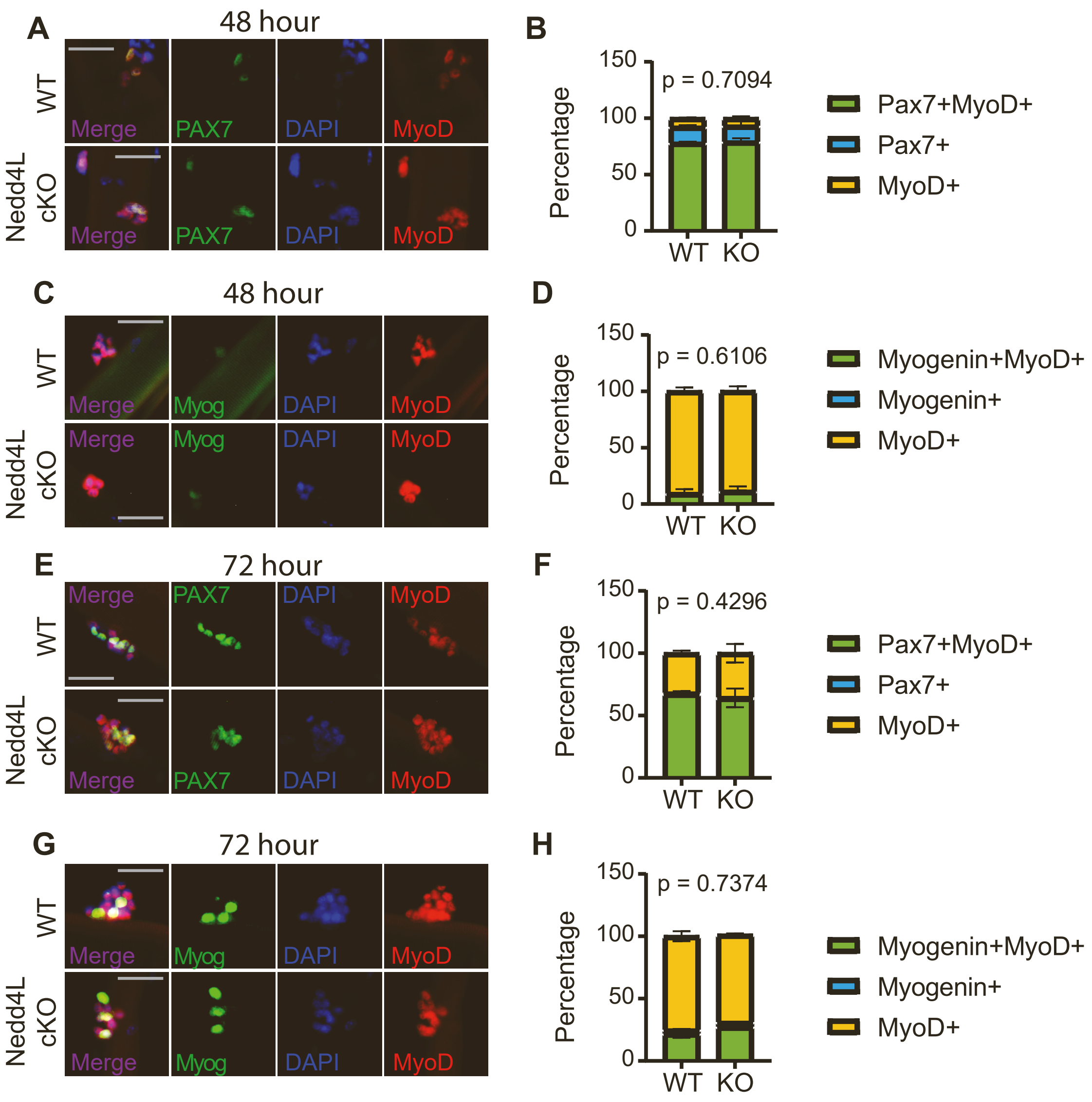

A
48 hour
WT
Nedd4L cKO
Merge
PAX7
DAPI
MyoD
B
Percentage
150
100
50
0
p = 0.7094
WT KO
Pax7+MyoD+
Pax7+
MyoD+
C
48 hour
Myog
D
p = 0.6106
Myogenin+MyoD+
Myogenin+
MyoD+
E
72 hour
F
p = 0.4296
G
72 hour
H
p = 0.7374

Figure S10

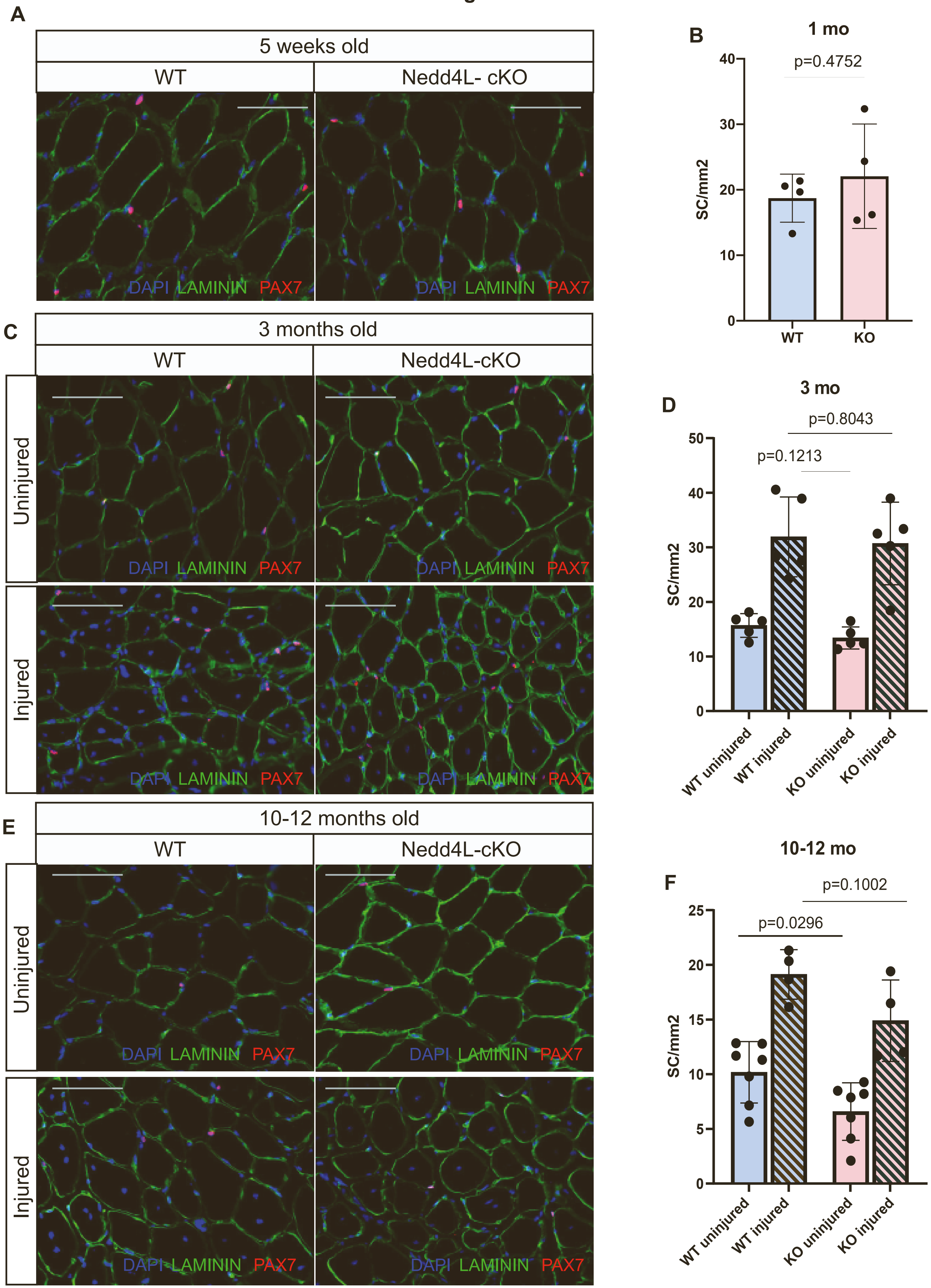

A
5 weeks old
WT
Nedd4L- cKO
DAPI LAMININ PAX7
B
1 mo
p=0.4752
SC/mm2
WT
KO
C
3 months old
WT
Nedd4L-cKO
Uninjured
Injured
D
3 mo
p=0.8043
p=0.1213
WT uninjured
WT injured
KO uninjured
KO injured
E
10-12 months old
F
10-12 mo
p=0.1002
p=0.0296

Supplemental information

Supplemental Figure Legends

Figure S1: **Expression of E3 ubiquitin ligases in MuSCs and Myoblasts, related to Figure 1**

A. Heatmap representing the significantly differentially expressed E3 ubiquitin ligases between muscle stem cells and primary myoblasts cultured for 3 days. Genes with a base mean count below 100 were removed.

B. Heatmap representing the Nedd4 E3 ubiquitin ligase family members expression between muscle stem cells and primary myoblasts cultured for 3 days.

C. Heatmap representing the significantly differentially expressed Nedd4 E3 ubiquitin ligase family members between muscle stem cells and primary myoblasts cultured for 3 days.

Figure S2: **Catalytic domain of Nedd4L is deleted in the genetic mouse model, related to Figure 2**

A. Diagram representing the genetic mouse model where loxp sites are present, surrounding exon 15, generated with Biorender.com. The Cre recombinase is linked to Pax7 expression, resulting in muscle stem cells having exon 15 deleted.

B. Bar graph representing the absolute expression in RPM from WT and Nedd4L-cKO MuSC RNA-Seq. N = 3; two tailed t-test, data presented as mean ± SD.

C. Heatmap of the top 100 differentially expressed genes between WT and Nedd4L-cKO MuSCs.

D. Volcano plot of the RNA stability of transcripts between WT and Nedd4L-cKO MuSCs.

Figure S3: **Genetic deletion of Nedd4L deregulates important pathways, related to Figure 3**

A. Bar graph of all of the significantly altered Hallmark pathways between the transcriptome of WT and Nedd4L-cKO MuSCs. Orange bars represent an upregulation in the Nedd4L-cKO; blue bars represent a downregulation.

B. Heatmaps of the expression of the genes associated with the G2M checkpoint, Myogenesis, and Il6 JAK STAT3 Signaling pathways, in the individual RNA-Seq replicates.

C. Bar graphs of selected representative genes in the G2M Checkpoint, Myogenesis, and Il6 JAK STAT3 Signaling pathways. N = 3; two tailed t-test, data presented as mean ± SD.

Figure S4: **Expression of myogenic factors and mKi67 in MuSCs, related to Figure 4**

A. Bar graph of the absolute expression in RPM of Pax7 from the WT and Nedd4L-cKO MuSC RNA-Seq. N = 3; two tailed t-test, data presented as mean ± SD.

B. Bar graph of the absolute expression in RPM of Myf5 from the WT and Nedd4L-cKO MuSC RNA-Seq. N = 3; two tailed t-test, data presented as mean ± SD.

C. Bar graph of the absolute expression in RPM of MyoD from the WT and Nedd4L-cKO MuSC RNA-Seq. N = 3; two tailed t-test, data presented as mean ± SD.

D. Bar graph of the absolute expression in RPM of Myogenin from the WT and Nedd4L-cKO MuSC RNA-Seq. N = 3; two tailed t-test, data presented as mean ± SD.

E. Bar graph of the absolute expression in RPM of Myf6 from the WT and Nedd4L-cKO MuSC RNA-Seq. N = 3; two tailed t-test, data presented as mean ± SD.

F. Bar graph of the absolute expression in RPM of mki67 from the WT and Nedd4L-cKO MuSC RNA-Seq. N = 3; two tailed t-test, data presented as mean ± SD.

G. Representative image of a WT TA cross section stained for PAX7 and Ki67. Scale bar = 40 µm.

H. Representative image of a Nedd4L-cKO cross section stained for PAX7 and Ki67. Scale bar = 40 µm.

I. Bar graph of the percentage of MuSCs double positive for Ki67 and PAX7. N = 3; two tailed t-test, data presented as mean ± SD.

J. Representative images of WT and Nedd4L-cKO TA cross sections, 21 days post CTX induced injury, stained for PAX7 and Ki67. Scale bar = 40 µm.

K. Bar graph of the percentage of MuSCs double positive for Ki67 and PAX7 in TA cross sections 21 days post CTX injury. N = 4; two tailed t-test, data presented as mean ± SD.

Figure S5: **Nedd4L-cKO MuSCs exhibit characteristics associated with G_{alert}, related to Figure 4**

A. Representative images of EDL associated MuSCs stained for Pax7 and phospho-S6 kinase, 0 hours post isolation. Scale bar = 25 µm.

B. Bar graph of the percentage of phospho-S6 kinase positive MuSCs, 0 hours post isolation. n = 3 mice per condition; two tailed t-test, data presented as mean ± SD.

C. Representative images of EDL associated MuSCs stained for Pax7 and phospho-S6 kinase, 6 hours post isolation. Scale bar = 25 µm.

D. Bar graph of the percentage of phospho-S6 kinase positive MuSCs, 6 hours post isolation. n = 3 mice per condition; two tailed t-test, data presented as mean ± SD.

E. Representative images of EDL associated MuSCs stained for Pax7 and phospho-S6 kinase, 24 hours post isolation. Scale bar = 25 µm.

F. Bar graph of the percentage of high expressing phospho-S6 kinase MuSCs, 24 hours post isolation. n = 3 mice per condition; two tailed t-test, data presented as mean ± SD.

G. Representative images of freshly isolated WT and Nedd4L-cKO MuSCs stained for phospho-S6 kinase. Scale bar = 40 µm.

H. Bar graph quantifying the percentage of high, medium, and low-level phospho-S6 kinase MuSCs. All cells were analyzed under the same imaging conditions and a threshold was established to assign the intensity level of the staining for high, medium, and low phospho-S6 kinase levels. n = 5 mice 2-month-old mice per condition; two way ANOVA, data presented as mean ± SD.

I. Representative images of EDL associated MuSCs stained for Pax7 and MyoD, 0 hours post isolation. Scale bar = 25 µm.

J. Bar graph of the percentage of MyoD+/Pax7+ MuSCs 0 hours post isolation. N = 3; two tailed t-test, data presented as mean ± SD.

K. Representative images of EDL associated MuSCs stained for Pax7 and MyoD, 6 hours post isolation. Scale bar = 25 µm.

L. Bar graph of the percentage of MyoD+/Pax7+ MuSCs 6 hours post isolation. N = 3; two tailed t-test, data presented as mean ± SD.

M. Representative images of EDL associated MuSCs stained for Pax7 and MyoD, 24 hours post isolation. Scale bar = 25 µm.

N. Bar graph of the percentage of MyoD+/Pax7+ MuSCs 24 hours post isolation. N = 3; two tailed t-test, data presented as mean ± SD.

Figure S6: Quiescent and activation genes are deregulated in Nedd4L-cKO MuSCs, related to Figure 5

A. Bar graph of the CHRDL2 protein levels of WT and Nedd4L-cKO MuSC proteomics. N = 4; two tailed t-test, data presented as mean ± SD.

B. Bar graph of the percentage of DCX expressing MuSCs associated to 0 hour post isolated EDL myofibers, from 6-month-old male mice. N = 3; two tailed t-test, data presented as mean ± SD.

C. Representative image of freshly isolated EDL myofibers stained for DCX and PAX7 from WT and Nedd4L-cKO 6-month-old mice. Scale bar = 25 µm.

D. Representative image of TA cross section from an uninjured WT mouse, stained for DCX and PAX7. Scale bar = 40 µm.

E. Representative image of TA cross sections from the contralateral TA of an injured WT mouse, stained for DCX and PAX7. Scale bar = 40 µm.

F. Representative image of TA cross sections from the contralateral TA of an injured Nedd4L-cKO mouse, stained for DCX and PAX7. Scale bar = 40 µm.

G. Bar graph of the percentage of DCX expressing MuSCs in uninjured TAs and contralateral TAs from injured mice. N = 4; two tailed t-test, data presented as mean ± SD.

H. Representative images of TA cross sections from the contralateral TA of an injured WT and Nedd4L-cKO mouse, stained for Ki67 and PAX7. Scale bar = 40 µm.

I. Bar graph of the percentage of Ki67 positive MuSCs in the contralateral TAs of an injured WT and Nedd4L-cKO mouse. N = 4; two tailed t-test, data presented as mean ± SD.

Figure S7: **Genes that are transcriptionally deregulated in the Nedd4L-cKO MuSCs show no difference in chromatin accessibility, related to Figure 6**

A. IGV tracks of ATAC-Seq peaks from WT and Nedd4L-cKO MuSCs of the NR1D1 gene.

B. IGV tracks of ATAC-Seq peaks from WT and Nedd4L-cKO MuSCs of the CHRDL2 gene.

C. IGV tracks of ATAC-Seq peaks from WT and Nedd4L-cKO MuSCs of the DCX gene.

D. IGV tracks of ATAC-Seq peaks from WT and Nedd4L-cKO MuSCs of the IL6 gene.

E. IGV tracks of ATAC-Seq peaks from WT and Nedd4L-cKO MuSCs of the CCL11 gene.

F. IGV tracks of ATAC-Seq peaks from WT and Nedd4L-cKO MuSCs of the B2M gene.

G. IGV tracks of ATAC-Seq peaks from WT and Nedd4L-cKO MuSCs of the MT1 gene.

H. IGV tracks of ATAC-Seq peaks from WT and Nedd4L-cKO MuSCs of the GAPDH gene. Data is presented as mean ± SD.

Figure S8: **Nedd4L-cKO myoblasts have no defect in survival or proliferation, related to Figure 7**

A. Representative images of EDL myofibers 72 hours post isolation and stained for MyoD and cleaved Caspase3 from WT, Nedd4L-cKO 6-8 weeks old mice. WT myofibers were treated with H_2O_2 as a positive control. Scale bar = 25µm.

B. Bar graph of the percentage of cleaved Caspase3 positive MuSCs that were associated to EDL myofibers 72 hours post isolation from WT and Nedd4L-cKO myofibers. WT myofibers were treated with H_2O_2 as a positive control. N = 3; two tailed t-test, data presented as mean ± SD.

C. Bar graph of the percentage of EDU positive primary myoblasts treated for 12 and 24 hours. N = 3; two tailed t-test, data presented as mean ± SD.

D. Representative images of WT and Nedd4L-cKO primary myoblasts treated with EDU for 12 and 24 hours. Scale bar = 25µm.

E. Western blot of Nedd4L, Pax7, MyoD and Gapdh as a loading control, from WT and Nedd4L-cKO primary myoblasts.

F. Western blot of Nedd4L, Pax7, Myogenin, and Gapdh as a loading control, from WT and Nedd4L-cKO myotubes cultured for 5 days in differentiation media. Data is presented as mean ± SD.

G. Representative images of freshly isolated EDL myofibers from WT and Nedd4L-cKO male 12-month-old mice, stained for Pax7.

H. Bar graph of the number of MuSCs from 0 hour post isolation EDL myofibers from WT and Nedd4L-cKO 12-month-old mice. N = 4 male mice; two tailed t-test, data presented as mean ± SD.

Figure S9: No differences in the ratio of the expression of myogenic factors in EDL associated myofibers are seen between WT and Nedd4L-cKO condition, related to Figure 7

A. Representative images of EDL associated MuSCs stained for Pax7 and MyoD, 48 hours post isolation. Scale bar = 25 µm.

B. Bar graph of the percentage of MuSCs positive for Pax7, MyoD, or double positive for Pax7 and MyoD 48 hours post isolation. N = 3; two way ANOVA, data presented as mean ± SD.

C. Representative images of EDL associated MuSCs stained for Myogenin and MyoD, 48 hours post isolation. Scale bar = 25 µm.

D. Bar graph of the percentage of MuSCs positive for Myogenin, MyoD, or double positive for Myogenin and MyoD 48 hours post isolation. N = 3; two way ANOVA, data presented as mean ± SD

E. Representative images of EDL associated MuSCs stained for Pax7 and MyoD, 72 hours post isolation. Scale bar = 25 µm.

F. Bar graph of the percentage of MuSCs positive for Pax7, MyoD, or double positive for Pax7 and MyoD 72 hours post isolation. N = 3; two way ANOVA, data presented as mean ± SD

G. Representative images of EDL associated MuSCs stained for Myogenin and MyoD, 72 hours post isolation. Scale bar = 25 µm.

H. Bar graph of the percentage of MuSCs positive for Myogenin, MyoD, or double positive for Myogenin and MyoD 72 hours post isolation. N = 3; two way ANOVA, data presented as mean ± SD

Figure S10: Nedd4L-cKO skeletal muscle does not have a regeneration defect, related to Figure 7

A. Representative image of a TA cross section from 5-week-old WT and Nedd4L-cKO mice. The cross sections were stained for PAX7, Laminin and counterstained with DAPI.

B. Bar graph of the average number of MuSCs per mm2 from the TA cross sections of 5-week-old WT and Nedd4L-cKO mice. n = 4 mice per condition; two tailed t-test, data presented as mean ± SD.

C. Representative image of uninjured and CTX injured TA cross sections from 3-month-old WT and Nedd4L-cKO mice. In the injured condition the TAs were allowed to regenerate for 21 days before the muscle was harvested. The cross sections were stained for PAX7, Laminin and counterstained with DAPI.

D. Bar graph of the average number of MuSCs per mm2 from the uninjured and injured TA cross sections of 3-month-old WT and Nedd4L-cKO mice. n = 5 mice per condition; two tailed t-test, data presented as mean ± SD.

E. Representative image of uninjured and CTX injured TA cross sections from 10–12-month-old WT and Nedd4L-cKO mice. In the injured condition the TAs were allowed to regenerate for 21 days before the muscle was harvested. The cross sections were stained for PAX7, Laminin and counterstained with DAPI.

F. Bar graph of the average number of MuSCs per mm2 from the uninjured and injured TA cross sections of 10–12-month-old WT and Nedd4L-cKO mice. n = 4-7 mice per condition; two tailed t-test, data presented as mean ± SD.
